# Atypical Pharmacodynamic Properties and Metabolic Profile of the Abused Synthetic Cannabinoid AB-PINACA: Potential Contribution to Pronounced Adverse Effects Relative to Δ^9^-THC

**DOI:** 10.3389/fphar.2018.01084

**Published:** 2018-09-26

**Authors:** Rachel D. Hutchison, Benjamin M. Ford, Lirit N. Franks, Catheryn D. Wilson, Azure L. Yarbrough, Ryoichi Fujiwara, Mark K. Su, Denise Fernandez, Laura P. James, Jeffery H. Moran, Amy L. Patton, William E. Fantegrossi, Anna Radominska-Pandya, Paul L. Prather

**Affiliations:** ^1^Department of Pharmacology and Toxicology, College of Medicine, University of Arkansas for Medical Sciences, Little Rock, AR, United States; ^2^Department of Biochemistry and Molecular Biology, College of Medicine, University of Arkansas for Medical Sciences, Little Rock, AR, United States; ^3^New York City Poison Control Center, New York, NY, United States; ^4^Jacobi Medical Center, Bronx, NY, United States; ^5^Translational Research Institute, University of Arkansas for Medical Sciences, Little Rock, AR, United States; ^6^PinPoint Testing, LLC, Little Rock, AR, United States

**Keywords:** CB1 receptors, G-protein coupled receptor, drug efficacy, drug toxicity, metabolite identification, pharmacokinetics, toxicology

## Abstract

Recreational use of marijuana is associated with few adverse effects, but abuse of synthetic cannabinoids (SCBs) can result in anxiety, psychosis, chest pain, seizures and death. To potentially explain higher toxicity associated with SCB use, we hypothesized that AB-PINACA, a common second generation SCB, exhibits atypical pharmacodynamic properties at CB1 cannabinoid receptors (CB1Rs) and/or a distinct metabolic profile when compared to Δ^9^-tetrahydrocannabinol (Δ^9^-THC), the principal psychoactive cannabinoid present in marijuana. Liquid chromatography tandem mass spectrometry (LC/MS) identified AB-PINACA and monohydroxy metabolite(s) as primary phase I metabolites (4OH-AB-PINACA and/or 5OH-AB-PINACA) in human urine and serum obtained from forensic samples. *In vitro* experiments demonstrated that when compared to Δ^9^-THC, AB-PINACA exhibits similar affinity for CB1Rs, but greater efficacy for G-protein activation and higher potency for adenylyl cyclase inhibition. Chronic treatment with AB-PINACA also results in greater desensitization of CB1Rs (e.g., tolerance) than Δ^9^-THC. Importantly, monohydroxy metabolites of AB-PINACA retain affinity and full agonist activity at CB1Rs. Incubation of 4OH-AB-PINACA and 5OH-AB-PINACA with human liver microsomes (HLMs) results in limited glucuronide formation when compared to that of JWH-018-M2, a major monohydroxylated metabolite of the first generation SCB JWH-018. Finally, AB-PINACA and 4OH-AB-PINACA are active *in vivo*, producing CB1R-mediated hypothermia in mice. Taken collectively, the atypical pharmacodynamic properties of AB-PINACA at CB1Rs relative to Δ^9^-THC (e.g., higher potency/efficacy and greater production of desensitization), coupled with an unusual metabolic profile (e.g., production of metabolically stable active phase I metabolites) may contribute to the pronounced adverse effects observed with abuse of this SCB compared to marijuana.

## Introduction

“K2” or “Spice” is a popular drug of abuse that is heavily marketed to young teens and first-time drug users as “safe” and/or “legal” marijuana” (Vardakou et al., 2010). Most K2 preparations consist of plant materials laced with a mixture of one or more SCB compounds possessing psychoactive properties similar to those produced by Δ^9^-tetrahydrocannabinol (Δ^9^-THC), the principal psychoactive compound found in marijuana (Fantegrossi et al., 2014). However, in contrast to the low incidence of adverse effects reported following use of marijuana, recreational abuse of SCBs can additionally result in anxiety, psychosis, chest pain, seizures and death (Ford et al., 2017b). Δ^9^-THC produces psychotropic actions by activating CB1 cannabinoid receptors (CB1Rs) in the CNS (Fujiwara and Egashira, 2004). Although structurally distinct from Δ^9^-THC, the abuse liability of both Δ^9^-THC and SCBs identified in K2 products results from their ability to potently and efficaciously activate CB1Rs (Brents and Prather, 2014). However, little is known concerning potential mechanisms by which SCBs induce more dangerous effects relative to marijuana.

AB-PINACA [(S)-N1(1-amino-3-methyl-1-oxobutan-2-yl)-1-pentyl-1H-indazole-3-carbox-amide] (Uchiyama et al., 2013; Wiley et al., 2015), is a popular “second generation” SCB that has been reported to produce dangerous adverse effects in human users (Thornton et al., 2015; Armenian et al., 2017). Several SCBs identified in K2 products to date exhibit distinct pharmacodynamic and pharmacokinetic properties relative to Δ^9^-THC that may contribute to the enhanced toxicity of these compounds (Fantegrossi et al., 2014). For example, in comparison to Δ^9^-THC that binds to CB1Rs with an affinity in the low nanomolar range (Showalter et al., 1996; Hess et al., 2016), many currently abused SCBs (e.g., 5F-PB-22, AK-B48, AB-FUBINACA, ADB-FUBINACA, JWH-122, and JWH-210) exhibit sub-nanomolar affinity for CB1Rs (Huffman et al., 2003, 2005; Banister et al., 2015; De Luca et al., 2016).

In addition to differences in CB1R affinity, most SCBs identified to date also regulate downstream intracellular effectors with higher potency and/or efficacy when compared to Δ^9^-THC (Ford et al., 2017b). For instance, although Δ^9^-THC produces potent CB1R-mediated activation of G-proteins and inhibition of adenylyl cyclase activity, it only acts as a partial agonist with reduced efficacy in these assays (Bonhaus et al., 1998; Canazza et al., 2016). In marked contrast to Δ^9^-THC, most SCBs reported (JWH-018, 5F-PB-22, MAM-2201, JWH-250, STS-135, XLR-11, AB-PINACA, EAM-2201, PB-22, AK-B48) not only regulate both of these effectors with high potency, but also act as full agonists to activate G-proteins and to inhibit adenylyl cyclase activity (Brents et al., 2011; Wiley et al., 2012, 2013; Costain et al., 2016; De Luca et al., 2016).

Finally, studies in our laboratory have shown that the pharmacokinetic profile of SCBs may also differ from that of Δ^9^-THC (Brents et al., 2012b). Δ^9^-THC is metabolized by cytochrome P450 enzymes CYP2C9 and CYP3A4 to a single major active metabolite (11-OH-Δ^9^-THC) with equivalent CB1R affinity (Compton et al., 1993) and slightly higher analgesic potency (Ford et al., 1977) when compared to the parent drug. In contrast, our group has reported that numerous phase one monohydroxylated metabolites of the SCBs JWH-073, JWH-018 and AM-2201 not only retain high affinity binding to CB1 and CB2Rs similar to that of the parent compound, but also act as partial and full agonists in both *in vitro* and *in vivo* assays (Brents et al., 2012a; Chimalakonda et al., 2012; Rajasekaran et al., 2013). Furthermore, several hydroxylated metabolites of the SCBs JWH-018, AM-2201, JWH-122, JWH-210, PB-22, MAM-2201, EAM-2201 and 5F-PB-22, not only retain higher *in vitro* affinity and activity than Δ^9^-THC, but also are the major phase I metabolites formed (Chimalakonda et al., 2012; Cannaert et al., 2016).

To potentially explain higher toxicity associated with SCB compared to Δ^9^-THC use, in this study we tested the hypothesis that AB-PINACA, a common second generation SCB, exhibits atypical pharmacodynamic properties at CB1Rs and/or a distinct metabolic profile when compared to Δ^9^-THC.

## Materials and Methods

### Materials

For human studies, ToxBox^®^ analytical test kits were provided by PinPoint Testing, LLC (Little Rock, AR, United States) to streamline sample preparation and testing procedures for SCBs, confounding drugs of abuse and other non-specified NPSs. ToxBox^®^ contained NIST-traceable, certified reference material for all standards and isotopically-labeled internal standards along with ISOLUTE^®^ SLE+ 96-well plates manufactured by Biotage (Charlotte, NC, United States). This newly developed kit identifies not only novel SCBs and associated metabolites, but also over 200 drugs and other NPSs that often confound such analytical tests. Optima-grade formic acid, acetonitrile, and methanol were purchased from Fisher Scientific (Fair Lawn, NJ, United States). Deionized water was purified to 18.2 MΩ⋅cm resistivity using the equivalent of a Millipore laboratory water purification system. All other chemicals and supplies were provided by Cerilliant (Round Rock, TX, United States), Cayman Chemical Company (Ann Arbor, Michigan, United States), Lipomed (Cambridge, MA), Biotage (Charlotte, NC, United States) or HemoStat Laboratories (Dixon, CA, United States). Blank defibrinated sheep blood or blank human blood void of drug contamination was used for all studies.

For all other *in vitro* studies, Δ^9^-THC was supplied by JM and the UAMS CDDR. AB-PINACA, 4OH-AB-PINACA, 5OH-AB-PINACA, JWH-018-M2, WIN-55,212-2, and CP-55,940 were obtained from Cayman Chemical Company (Ann Arbor, MI, United States). UDP-glucuronic acid (UDPGA), alamethicin and rimonabant were obtained from Sigma-Aldrich (St. Louis, MO, United States). All drugs were prepared as a stock solution in 100% DMSO at a concentration of 100 μM, divided into aliquots, and maintained at -4°C until use. [^3^H]CP-55,950 (168 Ci/mmol) and [^35^S]GTPγS (1250 Ci/mmol) were purchased from PerkinElmer (Boston, MA, United States). All other reagents were purchased from Fisher Scientific Inc. (Pittsburgh, PA, United States). Pooled HLMs were purchased from Corning Incorporated (Corning, NY, United States).

For *in vivo* studies, rimonabant was synthesized in the laboratory of Dr. Thomas E. Prisinzano at the University of Kansas School of Pharmacy (Lawrence, KS, United States), and provided to us as a generous gift. AB-PINACA, and its 4OH metabolite were obtained from Cayman Chemical Company (Ann Arbor, MI, United States). All cannabinoids were dissolved in a vehicle consisting of ethanol, emulphor and saline at a ratio of 1:1:18. Injections were administered via the intraperitoneal (IP) route at a constant volume of 0.1 cc/g. Rimonabant was administered at a dose of 10 mg/kg, 60 min before injection of AB-PINACA or its 4OH metabolite.

### Equipment

Supported liquid extraction (SLE) procedures were optimized for 96-wellplate processing on a PerkinElmer Zephyr G3 SPE Workstation (Waltham, MA, United States). Sample extracts were analyzed using an Agilent 1260 quaternary liquid chromatography system (Santa Clara, CA, United States) coupled to an Agilent 6420 tandem mass spectrometer (LC-MS/MS). Instrument control and data acquisition relied on MassHunter LC/MS Data Acquisition (VER B.08.00). Data analysis was performed using MassHunter Quantitative Analysis (VER B.07.01 SP2).

### Human Study Design

Results from de-identified human samples were used to demonstrate typical clinical results. Use of this material was approved by the Institutional Review Board of the University of Arkansas for Medical Sciences (Little Rock, AR, United States) (IRB #217584).

### Cell Culture

CHO-K1 cells stably expressing wild-type recombinant hCB1Rs (CNR1) were purchased from DiscoverRx Corporation (Fremont, CA, United States) and designated as CHO-hCB1. Transfected cells were cultured in HAM’s F-12 K media (ATCC, Manassas, VA, United States) that also contained 10% fetal calf serum (Gemini Bio Products, Sacramento, CA, United States), 1% penicillin/streptomycin (Invitrogen, Carlsbad, CA, United States), and 250 μg/mL of G418 (Sigma-Aldrich, St. Louis, MO, United States) in a humidified chamber at 37°C with 5% CO_2_. Cells were harvested when flasks reached approximately 70% confluence, and only cells from passages 4–15 were used in all experiments.

### Membrane Preparation

Established methods were used to prepare crude membrane homogenates of CHO-hCB1 cells (Prather et al., 2000). In brief, 70% confluent CHO-hCB1 cells were harvested, cell pellets were snap-frozen in liquid nitrogen, and stored at -80°C until use. At the time of membrane preparation, cell pellets were thawed on ice, pooled, and suspended in ice-cold homogenization buffer (50 mM HEPES, pH 7.4, 3 mM MgCl_2_, and 1 mM EGTA), before being subjected to 10 complete strokes using a 40 mL dounce glass homogenizer, and centrifuged at 40,000 *g* for 10 min at 4°C. Supernatants were discarded and pellets were re-suspended in ice-cold homogenization buffer, homogenized, and centrifuged as described previously two more times. Pellets were re-suspended in ice-cold 50 mM HEPES, pH 7.4 to achieve a final concentration of approximately 5 mg/mL and aliquoted for storage at -80°C. BCA^TM^ Protein Assay (Thermo Scientific, Rockford, IL, United States) was used to determine protein concentration.

### Competition Receptor Binding

Increasing concentrations (10^-11^ to 10^-5^ M) of experimental drug or vehicle were incubated with 0.2 nM of the non-selective CB1/CB2 agonist [^3^H]CP-55,940 in a final volume of 1 mL of binding buffer (50 mM Tris, 0.05% bovine serum albumin, 5 mM of MgCl_2_, pH 7.4) as described previously (Brents et al., 2011). Each binding assay contained 50 μg of membrane protein prepared from harvested CHO-hCB1 cells. Reactions were incubated for 90 min at room temperature. Non-specific binding was defined as binding observed in the presence of 10 μM of the non-radioactive non-selective CB1/CB2 ligand WIN-55,212-2. Reactions were terminated by rapid vacuum filtration through Whatman GF/B glass fiber filters followed by three washes with ice-cold binding buffer. Filters were then immediately placed into scintillation vials with 4 ml of Scintiverse^TM^-BD^®^ cocktail scintillation fluid (Fisher Scientific, Fair Lawn, NJ, United States). Samples were incubated overnight in scintillation fluid, vortexed and bound reactivity determined by employing a liquid scintillation spectrophotometer (Tri Carb 2100 TR Liquid Scintillation Analyzer, Packard Instrument Company, Meriden, CT, United States).

### [^35^S]GTPγS Binding

All [^35^S]GTPγS assays were conducted as described previously (Brents et al., 2012a) in buffer containing 20 mM HEPES, 100 mM NaCl, 10 mM MgCl_2_, 0.05% BSA, and 20 units/L of adenosine deaminase. Assays were performed in triplicate in a final volume of 1 ml with all reactions containing 0.1 nM [^35^S]GTPγS and increasing concentrations (10^-10^ to 10^-5^ M) of experimental drug or vehicle, and 10 μg of membrane protein prepared from CHO-hCB1 cells. Non-specific binding was defined by inclusion of 10 μM of non-radiolabeled GTPγS. The final concentration of DMSO in all reactions was 0.1%. Reactions were incubated at 30°C for 30 min and terminated by rapid vacuum filtration through Whatman GF/B glass fiber filters and followed by four 1 ml washes with ice-cold filtration buffer (20 mM HEPES, pH 7.4, 0.05% BSA). Filters were then immediately placed into scintillation vials with 4 ml of Scintiverse^TM^-BD^®^ cocktail scintillation fluid (Fisher Scientific, Fair Lawn, NJ, United States). Samples were incubated overnight in scintillation fluid, vortexed and bound reactivity determined by employing a liquid scintillation spectrophotometer (Tri Carb 2100 TR Liquid Scintillation Analyzer, Packard Instrument Company, Meriden, CT, United States).

### Adenylyl Cyclase Activity

All adenylyl cyclase assays were conducted using intact whole CHO-hCB1 cells as previously reported in (Ford et al., 2017a). In these assays, [^3^H]adenine is taken up by cells and converted to [^3^H]ATP, a substrate used by adenylyl cyclase to produce [^3^H]cAMP. Six million CHO-hCB1 cells were plated into 24-well plates to achieve approximately 80–90% confluency following overnight incubation in a humidified chamber at 37°C with 5% CO_2_. The following morning, growth media was removed and 0.5 mL of warm incubation media (DMEM with 0.9 g/L NaCl, 2.5 μCi/mL [^3^H]adenine and 0.5 mM IBMX) was added to each well of the 24-well plate. Following a 4-h incubation period at 37°C in a 5% CO_2_ incubator, media was removed and the plate was briefly floated on an ice water bath while 0.5 mL of an assay mix was quickly added per well of cells in triplicate. The assay mix consisted of a Krebs Ringer HEPES buffered saline solution containing 0.5 mM IBMX, 10 μM forskolin and increasing concentrations (10^-10^ to 10^-5^ M) of experimental drug or vehicle. The final concentration of DMSO in all reactions was 0.1%. Plates were then transferred to a 37°C water bath for a 15 min incubation. Reactions were terminated by addition of 50 μL of 2.2 N HCl. Intracellular [^3^H]cAMP was separated by column chromatography employing acidic alumina. Four mL of the final eluent was added to 10 mL of Scintiverse-BD Cocktail Scintillation Fluid (Fisher Scientific, Fair Lawn, NJ, United States) and radioactivity was immediately measured employing liquid scintillation spectrophotometry (Tri Carb 2100 TR Liquid Scintillation Analyzer, Packard Instrument Company, Meriden, CT, United States).

### Glucuronidation of AB-PINACA and JWH-018 Metabolites in HLMs

A typical incubation mixture (30 μl of total volume) contained 100 mM Tris-HCl (pH 7.4), 5 mM MgCl_2_, 3 mM UDPGA, 80 μg/ml alamethicin, 50 μg HLMs, 5 mM saccharolactone, and 50 μM 4OH- or 5OH-AB-PINACA. To compare the clearance of 4OH-AB-PINACA and JWH-018-M2 through glucuronidation, 5 μM 4OH-AB-PINACA or JWH-018-M2 were incubated with HLMs in the same assay condition. After incubation at 37°C for 90 min, reactions were terminated by the addition of 30 μl cold ethanol. Following removal of the protein by centrifugation at 12,000 × *g* for 10 min, a 5-μl portion of the sample was subjected to UPLC. All incubations were performed in triplicate.

Parent compounds and glucuronidated metabolites were identified by the ACQUITY UPLC System with a UV detector (Milford, MA, United States). The mobile phases were 0.1% acetic acid (A) and 100% methanol (B), and the flow rate was 0.5 mL/min with an elution gradient of 100% A (0–0.2 min), a linear gradient from 100% A to 25% A-75% B (0.2–5 min), and 100% B (5–7 min). The column was re-equilibrated at initial conditions for 2.5 min between runs. The elution was monitored at 300 nm. The retention times of 4OH-AB-PINACA, 5OH-AB-PINACA, 4OH-AB-PINACA glucuronide, and JWH-018-M2 were 3.989, 3.994, 3.646, and 5.391 min.

### Supported Liquid Extraction of Standards, Quality Control Material, and Specimens

All blood calibration standards, QC material, and unknown samples were processed identically by mixing 0.25 ml of blank blood or unknown specimen in appropriate wells at 900 rpm for 15 min, pretreating with 0.25 mL of 0.5 M ammonium hydroxide, and then mixing for another 15 min at 900 rpm. Samples were then loaded, under gentle vacuum or positive pressure, onto a 400 mg ISOLUTE^®^ SLE+ 96-wellplate. Samples were allowed to equilibrate for 5 min before extracting under gravity with two fractions of 900 μL of ethyl acetate. All sample extracts were evaporated to dryness under a gentle flow of nitrogen and reconstituted in 100% methanol (100 μl).

### Liquid Chromatography Tandem Mass Spectrometry

Analytical procedures used 5 μl injections on a 2.6 μm Phenomenex Kinetex Phenyl Hexyl (50 × 4.6 mm) LC column heated to 35°C. Analytes were resolved at 0.5 mL/min using mobile phase A (10 mM ammonium formate in ultrapure 18.2 MΩ⋅cm water) and mobile phase B (0.1% formic acid in methanol). Analytes are resolved using a gradient starting at 95% aqueous (Mobile Phase A) and ramping to 0% aqueous over 4 min, and holding constant for 1 min. The gradient returned to initial conditions over 0.1 min and equilibrated for an additional 1.9 min. The total run time including column equilibration period between injections was 7.0 min. Specific mass spectrometer and analyte parameters are provided in **Supplementary Tables S2, S3**.

### Animals

Adult male CD1 mice (Charles River Laboratory, Wilmington, MA, United States) were housed three subjects per cage (15.2 cm × 25.4 cm × 12.7 cm) in a temperature-controlled room in a vivarium accredited by the Association for Assessment and Accreditation of Laboratory Animal Care, and given *ad libitum* food (Laboratory Rodent Diet no. 5001; PMI Feeds, St. Louis, MO, United States) and water. The vivarium was maintained at 22°C ± 2°C and 45–50% humidity, with lights set to a 12-h light/dark cycle (lights on at 6:00 AM, CST). Testing was performed during the inactive phase (8:00 AM to 4:00 PM). All procedures were carried out in accordance with the Guide for Care and Use of Laboratory Animals as adopted and promulgated by the National Institutes of Health (NIH). The experimental protocol has been approved by the Institutional Animal Care and Use Committee (IACUC) at the University of Arkansas for Medical Sciences (UAMS).

### Cannabinoid-Elicited Hypothermia

Mice were habituated to handling and weighing prior to initiation of experimental procedures. A digital thermometer (model BAT-12, PhysiTemp, Clifton, NJ, United States) equipped with a Ret-3 mouse probe (model 50314, Stoelting Co., Dale, IL, United States) was lubricated and inserted approximately 2 cm into the rectum. Stable temperatures were obtained within ∼6 s. Rectal temperature data were sampled immediately before injection, and at 10, 30, 60, 120, 240, and 360 min after injection.

### Data Analysis

Curve-fitting was conducted utilizing GraphPad Prism^®^ v6.0b (GraphPad Software, Inc.; San Diego, CA, United States). Non-linear regression for one-site competition was used to determine the IC_50_ for competition receptor binding. The Cheng-Prusoff equation (Cheng and Prusoff, 1973) was employed to convert the experimental IC_50_ values obtained from competition receptor binding experiments to *K*_i_ values (a quantitative measure of receptor affinity). Curve fitting of concentration-effect curves *via* non-linear regression was also employed to determine the ED_50_ or IC_50_ (measures of potency) and *E*_max_ or *I*_max_ (measures of efficacy) for the [^35^S]GTPγS and adenylyl cyclase experiments, respectively. All dissociation constants and measurements of potency were converted to pK_i_, pEC_50_, or pIC_50_ values by taking the negative log of each value so that parametric tests could be used for statistical comparisons. A one-way ANOVA, followed by Tukey’s Multiple Comparison *post hoc* test, was used to determine statistical significance (*P* < 0.05). For animal studies, temperature data are shown as mean values (±SEM) for groups of subjects at each drug dose and time point. For statistical analyses, only the highest dose of AB-PINACA or its 4OH- metabolite (with and without rimonabant pretreatment) were considered. The lowest temperatures recorded for each subject within each group (regardless of when after drug administration it occurred) were compared to vehicle data using a one-way ANOVA and Tukey’s HSD *post hoc* test. Significance was judged at *P* < 0.05.

## Results

**FIGURE 1:**
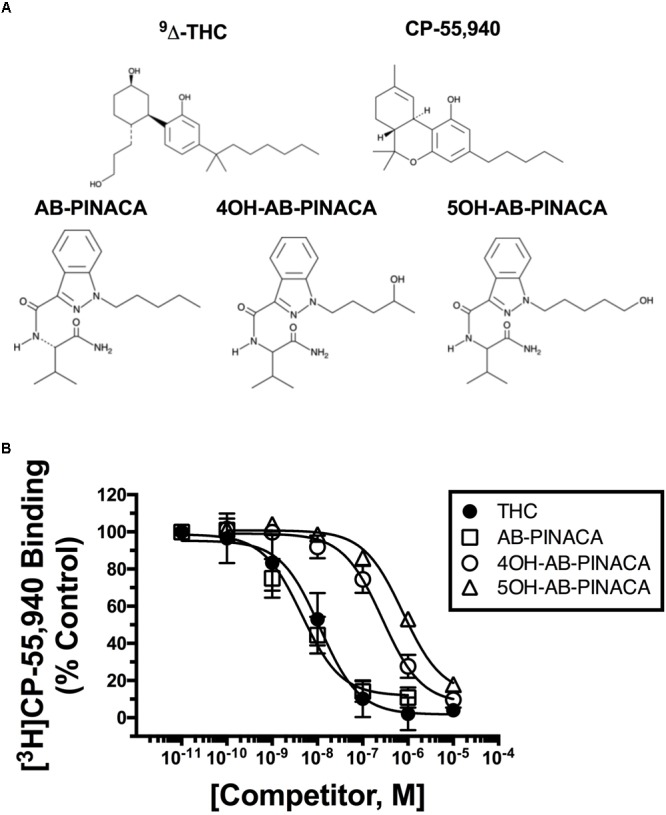
**(A)** Structures of drugs used in this study. **(B)** Competition receptor binding in CHO-hCB1 cell membranes to compare CB1R affinity of SCBs examined. The affinity of AB-PINACA (open squares) for hCB1Rs is similar to that of the well characterized Δ^9^-THC (filled circles). Although exhibiting reduced hCB1R affinity compared to the parent compound, both hydroxylated AB-PINACA metabolites (4OH-AB-PINACA, open circles and 5OH-AB-PINACA, open triangles) nevertheless maintain nanomolar affinity for hCB1Rs. Specific binding was determined by incubating 0.2 nM of [^3^H]CP-55,950 with increasing concentrations of the indicated drugs and 50 μg of membranes prepared from CHO-hCB1 cells (see section “Materials and Methods”). Data points presented are the mean ± SEM of [^3^H]CP-55,940 binding in the presence of test compounds. The Cheng–Prusoff equation (Cheng and Prusoff, 1973) was used to convert the experimental IC_50_ values obtained from competition receptor binding experiments to *K*_i_ values, a quantitative measure of receptor affinity. The mean ± SEM and statistical comparison of pK_i_ values calculated for each SCB are presented in **Table 2**.

It has previously been reported by incubation with pooled human hepatocytes that AB-PINACA is metabolized to two primary phase I oxidative metabolites, 4OH- and 5OH-AB-PINACA (**Figure 1A**) (Wohlfarth et al., 2015). The UAMS CDDR provides surveillance of drug exposure to SCBs and other NPSs. To confirm the predicted metabolic profile for AB-PINACA in human urine and serum for forensically obtained samples, results from the CDDR clinical database were searched which provided one clinical specimen where both clinical samples and the actual product purportedly consumed were available for testing. Analytical results for the product and clinical specimens are provided in **Table 1**. Patient demographics and case information for these samples were unavailable. The purported product consumed by Patient 002 contained both AB-PINACA and AB-CHMINACA. Urine analysis showed the presence of AB-PINACA, and metabolites of AB-PINACA. In the analysis presented here, the 4OH- and 5OH- metabolites were not resolved in this chromatographic procedure, so the total combined urinary concentration is reported as 33.1 ng/ml. Analysis in blood did not detect metabolites of AB-PINACA. Metabolites of AB-CHMINACA (M1A and M3A) were detected in both urine (approximately 40 ng/ml) and blood (1.5 and 13.6 ng/ml). Interestingly, parent drug or downstream metabolites of AB-FUBINACA, MAB-CHMINACA, and UR-144 were detected in blood and/or urine. While speculative, detection of these SCBs likely represent previous exposures. No other confounding drugs or NPSs from a panel of over 200 chemicals were detected in either the product seized or the patient’s urine (see **Supplementary Table S1** for analyte list).

**Table 1:** Analytical results of forensic clinical samples.

Sample	Analyte(s) detected	Concentration (ng/mL)	Confounding drugs detected
Product	AB-PINACA	N/A	None detected
	AB-CHMINACA	N/A	
Urine	AB-FUBINACA	1.1	None detected
	AB-PINACA	0.9	
	AB-CHMINACA M1A	35.4	
	AB-CHMINACA M3A	37.8	
	AB-PINACA-(5OH/4OH)	33.1	
Serum	AB-FUBINACA	^#^P	Not tested^∗^
	AB-PINACA	1.2	
	MAB-CHMINACA	11.3	
	AB-CHMINACA M1A	1.5	
	AB-CHMINACA M2	13.6	
	MAB-CHMINACA M3	6.0	
	UR-144-pentanoic acid degradant	5.4	

*^#^Detected but not quantified, ^∗^sufficient volume was not available for testing.*

Initial *in vitro* studies were next conducted to determine the affinity of Δ^9^-THC, AB-PINACA and primary metabolites 4OH- and 5OH-AB-PINACA (**Figure 1A**) for human CB1Rs stably expressed in CHO-hCB1 cells (**Figure 1B** and **Table 2**). AB-PINACA produced concentration-dependent and complete displacement of the non-selective CB1/CB2 cannabinoid radioligand [^3^H]CP-55,940 from hCB1Rs with an apparent affinity (*K*_i_) of 4.0 nM (open squares). The affinity of AB-PINACA for hCB1Rs is similar to that of the well characterized, high affinity non-selective CB1/CB2 agonist CP-55,940 (0.92 nM; not shown) (Ford et al., 2017a) and Δ^9^-THC (9.6 nM; filled circles). Importantly, although exhibiting reduced hCB1R affinity when compared to the parent compound (*P* < 0.05; **Table 2**), both hydroxylated AB-PINACA metabolites nevertheless maintain nanomolar affinity for hCB1Rs (4OH-AB-PINACA, 159 nM; open circles and 5OH-AB-PINACA, 452 nM; open triangles).

**Table 2:** Affinity, potency and efficacy values (*in vitro* studies).

	[^3^H]CP-55,940 binding			[^35^S]GTPγS binding				cAMP accumulation			
Ligand	*pK*_i_	*K*_i_ (nM)	*N*	pEC_50_	EC_50_ (nM)	*E*_MAX_	*N*	pIC_50_	IC_50_ (nM)	*I*_MAX_	*N*
CP-55,940	9.07 ± 0.13^a,†^	0.92^†^	3	8.03 ± 0.18^a^	11.0	64.0 ± 3.8^a^	3	7.85 ± 0.16^a,b,c^	16.3	86.7 ± 6.4^a^	3
^9^Δ-THC	8.10 ± 0.17^a^	9.6	4	8.05 ± 0.14^a^	12.5	39.0 ± 2.7^b^	7	7.35 ± 0.20^a,c^	68.8	91.1 ± 2.3^a^	6
AB-PINACA	8.75 ± 0.25^a^	4.0	7	8.06 ± 0.17^a^	12.8	71.9 ± 5.8^a^	10	8.65 ± 0.19^b^	3.9	72.0 ± 2.1^b^	6
4OH-AB-PINACA	6.82 ± 0.09^b^	159	3	6.09 ± 0.12^b^	952	92.6 ± 13.4^a^	5	6.89 ± 0.17^c^	141	87.0 ± 2.9^a^	3
5OH-AB-PINACA	6.35 ± 0.06^b^	452	3	5.55 ± 0.08^c^	2742	100 ± 16.8^a^	3	–	–	–	

*E_*MAX*_ and I_*MAX*_ values are presented as the percent (%) of basal activity in the presence of vehicle. ^*a,b,c*^pK_*i*_, pEC_*50*_, and pIC_*50*_ values designated by different letters are significantly different from values within the same column; P < 0.05, one-way ANOVA, Tukey’s post hoc test. ^†^pK_*i*_ and K_*i*_ values for CP-55,940 were obtained from that previously published in Ford et al. (2017a).*

**FIGURE 2:**
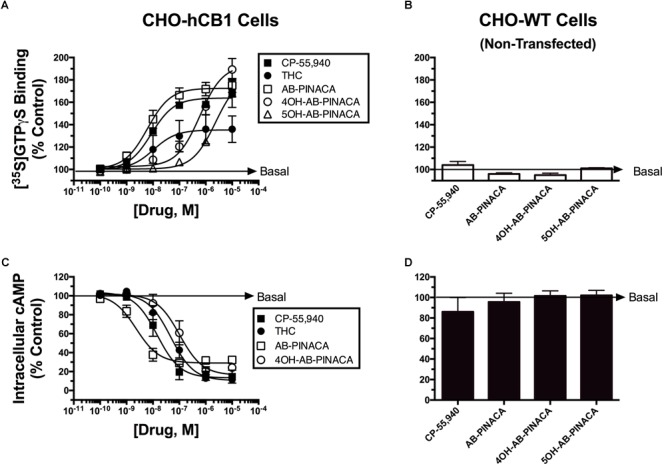
Quantification of intrinsic activity of SCBs at hCB1Rs by examining modulation of G-protein and adenylyl cyclase activity in CHO-hCB1 and non-transfected wild-type (CHO-WT) cells. G-protein activation in CHO-hCB1 **(A)** or CHO-WT **(B)** cell membranes by SCBs to compare CB1R potency and efficacy. Membranes (50 μg) prepared from CHO-hCB1 cells were incubated in the presence of 0.1 nM [^35^S]GTPγS with increasing concentrations (10^-10^ to 10^-5^ M) of CP-55,950 (filled squares), Δ^9^-THC (filled circles), AB-PINACA (open squares), 4OH-AB-PINACA (open circles) or 5OH-AB-PINACA (open triangles) (see section “Materials and Methods”). Data points presented are the mean ± SEM of basal G-protein activity in the presence of test compounds. Curve fitting of concentration-effect curves *via* non-linear regression was employed to determine the ED_50_ (measure of potency) and *E*_max_ (measure of efficacy) for G-protein activation by each agonist. The mean ± SEM and statistical comparison of ED_50_ and *E*_max_ values calculated for each SCB are presented in **Table 2**. Adenylyl cyclase activity in whole intact CHO-hCB1 **(C)** or CHO-WT **(D)** cells by SCBs to compare CB1R potency and efficacy. Following incubation of CHO-hCB1 cells with [^3^H]adenine for 4 h and washout, increasing concentrations (10^-10^ to 10^-5^ M) of CP-55,950 (filled squares), Δ^9^-THC (filled circles), AB-PINACA (open squares) or 4OH-AB-PINACA (open circles) were added to cells for 15 min (see section “Materials and Methods”). Data points presented are the mean ± SEM of basal intracellular cAMP in the presence of test compounds. Curve fitting of concentration-effect curves *via* non-linear regression was employed to determine the IC_50_ (measure of potency) and *I*_max_ (measure of efficacy) for adenylyl cyclase modulation by each agonist. The mean ± SEM and statistical comparison of IC_50_ and *I*_max_ values calculated for each SCB are presented in **Table 2**.

Subsequent experiments were performed to determine the intrinsic activity of AB-PINACA and its primary metabolites at CB1Rs (**Figure 2** and **Table 2**). The CB1 cannabinoid receptor is a G-protein coupled receptor that produces intracellular effects via interaction with the Gi/Go-subtype of G-proteins (Dalton et al., 2009). Upon binding to CB1Rs, agonists produce activation of G-proteins that can be quantified in membrane preparations by measuring increases in agonist-induced binding of [^35^S]GTPγS, a non-hydrolyzable GTP analog (Harrison and Traynor, 2003). Therefore, as an initial measure of intrinsic activity, the ability of increasing concentrations of CP-55,940 (filled squares), Δ^9^-THC (filled circles), AB-PINACA (open squares) and primary metabolites 4OH- (open circles) and 5OH-AB-PINACA (open triangles) to increase [^35^S]GTPγS binding in CHO-hCB1 membranes was examined (**Figure 2A**). CP-55,940 and Δ^9^-THC both act as hCB1 agonists, producing potent (e.g., 11.0 and 12.5 nM, respectively) activation of G-proteins. However, as reported previously (Brents et al., 2011, 2012b), CP-55,950 acts as a full hCB1 agonist producing maximal G-protein activation (64.0 ± 3.8%) compared to the partial agonist Δ^9^-THC which exhibits reduced efficacy in this assay (39.0 ± 2.7%) (*P* < 0.05; **Table 2**). AB-PINACA also acts as a full hCB1 agonist, producing a concentration-dependent increase in [^35^S]GTPγS binding with a potency of 12.8 nM and efficacy of 71.9 ± 5.8%. Remarkably, although acting with reduced potency, both AB-PINACA metabolites also retain full agonist activity, activating G-proteins with similar efficacy to that produced by the full agonists CP-55,940 and AB-PINACA.

To provide a second measure of intrinsic activity, the ability of cannabinoids to inhibit activity of adenylyl cyclase, an intracellular effector immediately downstream from G-protein activation (Dalton et al., 2009), was examined (**Figure 2C** and **Table 2**). The affinity of 4OH-AB-PINACA and 5OH-AB-PINACA for hCB1 receptors was determined to be 159 and 452 nM, respectively. Due to the relatively low affinity of the 5OH- relative to the 4OH-metabolite for hCB1Rs, for the adenylyl cyclase assays, we chose to focus studies by examining effects of the potentially more physiologically relevant 4OH-AB-PINACA compound. In this assay, measuring modulation of adenylyl cyclase activity (a downstream amplified response relative to G-protein activation), both CP-55,940 (filled squares) and Δ^9^-THC (filled circles) act as potent (16.3 and 68.8 nM, respectively) and fully efficacious (86.7 ± 6.4% and 91.1 ± 2.3% inhibition, respectively) hCB1R agonists. Interestingly, AB-PINACA (open squares) inhibits adenylyl cyclase activity to lower intracellular cAMP levels with reduced efficacy (72.0 ± 2.1%), but higher potency (3.9 nM) when compared to CP-55,940 and Δ^9^-THC (*P* < 0.05; **Table 2**). Most importantly, although acting with lower potency (141 nM), the AB-PINACA metabolite (4OH-AB-PINACA; open circles) exhibits higher efficacy (87.0 ± 2.9%) in the adenylyl cyclase assay when compared to the parent compound AB-PINACA (*P* < 0.05; **Table 2**).

To verify that both G-protein-dependent effects observed for the cannabinoid ligands examined are due to specific interaction with hCB_1_Rs, G-protein activation and modulation of adenylyl cyclase activity studies were conducted in CHO cells devoid of hCB_1_Rs (CHO-WT). These studies showed that no cannabinoid ligand examined either activates G-proteins (**Figure 2B**) or inhibits adenylyl cyclase activity (**Figure 2D**).

**FIGURE 3:**
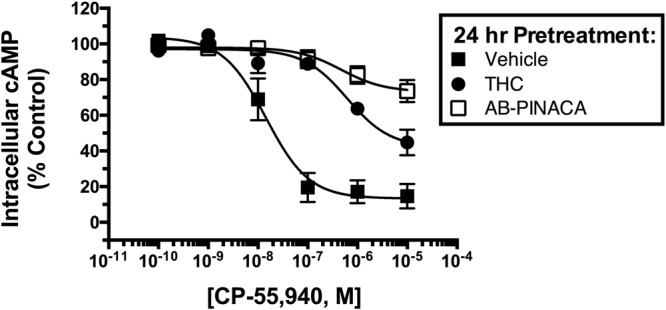
Comparison of CB1R desensitization produced by chronic SCB treatment. CHO-hCB1 cells were exposed to vehicle (filled squares) or a receptor saturating concentration of Δ^9^-THC (1 μM, filled circles) or AB-PINACA (1 μM, open squares) for 24 h. Following chronic exposure, cells were washed extensively to remove residual drug and the ability of increasing concentrations (10^-10^ to 10^-5^) CP-55,940 to modulate adenylyl cyclase activity was examined. Data points presented are the mean ± SEM of basal intracellular cAMP in the presence of CP-55,940 following chronic treatment with the respective vehicle or drug. Curve fitting of concentration-effect curves *via* non-linear regression was employed to determine the IC_50_ (measure of potency) and *I*_max_ (measure of efficacy) for adenylyl cyclase modulation by CP-55,940 following chronic exposure to vehicle or each agonist. The mean ± SEM and statistical comparison of IC_50_ and *I*_max_ values calculated for each SCB are presented and discussed in the Results section.

Studies were next conducted to compare the adaptive effects produced by chronic treatment with Δ^9^-THC versus AB-PINACA (**Figure 3**). Upon prolonged agonist binding to GPCRs, specific intracellular serine and threonine residues of activated GPCRs are phosphorylated, a second protein β-arrestin is then recruited to phosphorylated GPCRs, interfering with G protein coupling and signaling (e.g., receptor desensitization) (Delgado-Peraza et al., 2016). To compare desensitization of hCB1Rs produced by AB-PINACA relative to Δ^9^-THC, CHO-hCB1 cells were exposed to a receptor saturating concentration (1 μM) of each cannabinoid or vehicle for 24 h. Following chronic exposure, cells were washed extensively to remove residual drug and the ability of CP-55,940 to modulate adenylyl cyclase activity was examined. In intact whole CHO-hCB1 cells exposed to vehicle for 24 h (filled squares), the hCB1 full agonist CP-55,940 produces potent (28.8 nM) and efficacious (86.7 ± 6.4%) inhibition of adenylyl cyclase activity as previously described (see **Figure 2C** and **Table 2**). Chronic treatment of CHO-hCB1 cells with AB-PINACA (open squares) and Δ^9^-THC (filled circles) reduces both the potency (983 and 611 nM, respectively) and efficacy (28.0 ± 6.0% vs. 58.0 ± 8.0%, respectively) of CP-55,940 to inhibit adenylyl cyclase activity compared to vehicle treatment (*P* < 0.05). However, prolonged exposure to AB-PINACA produces a greater reduction (*P* < 0.05) in the efficacy of CP-55,940-mediated adenylyl cyclase inhibition than does similar treatment with Δ^9^-THC.

**FIGURE 4:**
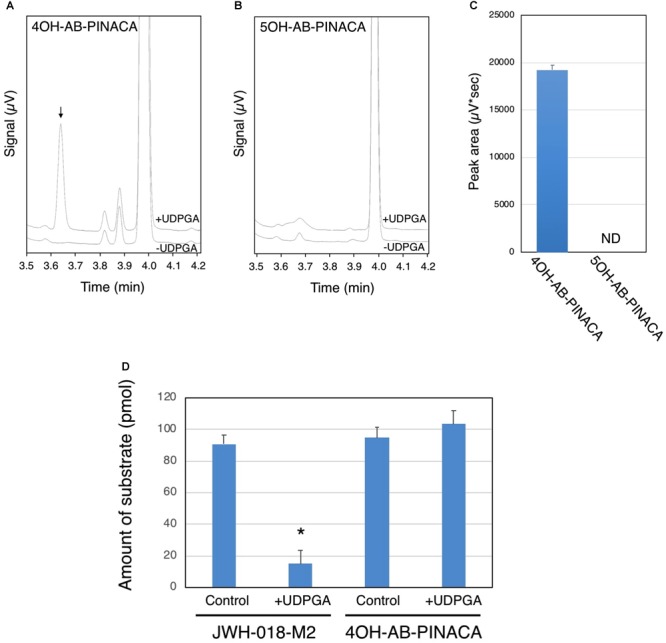
Glucuronidation of 4OH- and 5OH-AB-PINACA in human liver microsomes (HLMs). 4OH-AB-PINACA **(A)** and 5OH-AB-PINACA **(B)** were incubated for 90 min with HLMs in the presence or absence of UDPGA. The terminated reaction mixtures were subjected to the UPLC analysis and the eluent was monitor with a UV detector at 300 nm and chromatograms presented. **(C)** The peak area of the glucuronide was estimated from the triplicate determinations. The data are presented as mean value ± SD. ND, not detected. **(D)** Comparison of glucuronidation of JWH-018-M2 and 4OH-AB-PINACA with HLMs. JWH-018-M2 and 4OH-AB-PINACA (5 μM) were incubated for 90 min with HLMs in the presence or absence of UDPGA. The terminated reaction mixtures were subjected to the UPLC analysis and the eluent was monitor with a UV detector at 300 nm. The remaining amount of the substrates was quantified by using standard curves of JWH-018-M2 and 4OH-AB-PINACA. The data was shown as mean value ± SD. ^∗^*P* < 0.001.

UDP-glucuronosyltransferases (UGTs) are microsomal enzymes that glucuronidate various xenobiotics including drugs such as SCBs. In this study, studies were next conducted to evaluate the ability of HLMs to glucuronidate 4OH-AB-PINACA and 5OH-AB-PINACA, which are the major oxidized metabolites of AB-PINACA (**Figure 4**). After 90 min incubation of the tested substrates with HLMs, the majority of the substrates remain unreacted (**Figures 4A,B**). A unique peak is observed when 4OH-AB-PINACA is incubated with HLMs in the presence of UDPGA, a co-substrate of UGTs (**Figures 4A,C**). In contrast, no such peak is observed with 5OH-AB-PINACA (**Figures 4B,C**).

It has been reported that humans excrete monohydroxy metabolites of AB-PINACA (Wohlfarth et al., 2015). To help understand the potential importance of glucuronidation, the hepatic clearance of 4OH-AB-PINACA was compared to that of JWH-018-M2, a major hydroxylated metabolite of the first generation SCB JWH-018, via glucuronidation. For these studies, both compounds were incubated with HLMs for 90 min in the presence and absence of UDPGA. After the 90 min incubation, the remaining amount of 4OH-AB-PINACA and JWH-018-M2 was quantified by the UPLC analysis (**Figure 4D**). The remaining amount of JWH-018-M2 is significantly lower when incubated with HLMs in the presence of UDPGA, indicating that the majority of JWH-018-M2 is biotransformed to its glucuronide. In contrast, such disappearance of substrate is not observed with 4OH-AB-PINACA.

**FIGURE 5:**
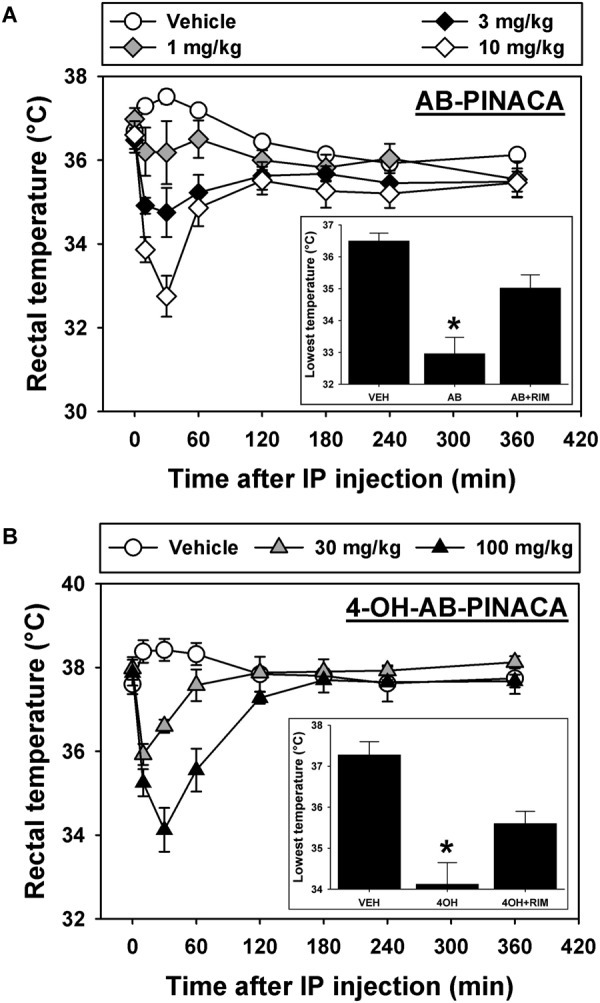
**(A)** Effects of intraperitoneal administration (i.p.) of vehicle (open circles), 1.0 mg/kg (gray diamonds), 3.0 mg/kg (black diamonds) or 10.0 mg/kg (open diamonds) AB-PINACA on rectal temperature (in°C) as a function of time. Points represent group means, ±SEM, and any points without error bars indicate that the variability is contained within the point. *Inset –* Lowest rectal temperatures recorded following injection of vehicle, 10.0 mg/kg AB-PINACA, or 10.0 mg/kg rimonabant administered 60 min before 10.0 mg/kg AB-PINACA. Bars represent group means, ±SEM, and asterisks represent significant differences from vehicle. The asterisk in the panel **A** inset indicates that the hypothermic effect produced by AB-PINACA is significantly different (*P* < 0.05) from that observed following injection of vehicle and from that observed following administration of rimonabant with AB-PINACA. **(B)** Effects of vehicle (open circles), 30.0 mg/kg (gray triangles) or 100.0 mg/kg (black triangles) 4OH-AB-PINACA on rectal temperature (in°C) as a function of time. All graph properties as described in **Figure 1**. *Inset –* Lowest rectal temperatures recorded following injection of vehicle, 100.0 mg/kg 4OH-AB-PINACA, or 10.0 mg/kg rimonabant administered 60 min before 100.0 mg/kg 4OH-AB-PINACA. All graph properties as described in **Figure 1**. The asterisk in the panel **B** inset indicates that the hypothermic effect produced by 4OH-AB-PINACA is significantly different (*P* < 0.05) from that observed following injection of vehicle and from that observed following administration of rimonabant with 4OH-AB-PINACA.

Finally, experiments were conducted to determine if the primary active AB-PINACA metabolite 4OH-AB-PINACA retained cannabimimetic activity in animals (**Figure 5**). Intraperitoneal administration (i.p.) of the cannabinoid vehicle produces a small (∼1°C) and transient increase in rectal temperature which resolves over approximately 2 h (**Figure 5A**, open circles). In contrast, the lowest tested dose of AB-PINACA (**Figure 5A**, gray diamonds) prevents the typical injection-elicited increase in rectal temperature, while higher doses of 3.0 (**Figure 5A**, black diamonds) and 10.0 mg/kg (**Figure 5A**, open diamonds) elicit dose- and time-dependent hypothermic effects. Statistical analysis of the lowest rectal temperature achieved following injection of vehicle, 10.0 mg/kg AB-PINACA, or 10.0 mg/kg rimonabant prior to 10.0 mg/kg AB-PINACA detect a significant difference among groups (**Figure 5A**, inset; *F* = 19.157, *P* < 0.05). Importantly, this difference is driven by AB-PINACA alone eliciting a hypothermic effect which was significantly different from that observed following injection of the vehicle (*q* = 8.715, *P* < 0.05) and from that observed following administration of rimonabant with AB-PINACA (*q* = 5.071, *P* < 0.05). These data demonstrate dose-dependent hypothermic effects of AB-PINACA which are mediated by agonist actions at CB1Rs in the mouse.

In studies designed to determine the biological effects of 4OH-AB-PINACA, vehicle injection again induces a small and short-lived increase in rectal temperature which resolves over approximately 2 h (**Figure 5B**, open circles), while administration of 30.0 (**Figure 5B**, gray triangles) and 100.0 mg/kg 4OH-AB-PINACA (**Figure 5B**, black triangles) elicits dose- and time-dependent hypothermic effects. Statistical analysis of the lowest rectal temperature achieved following injection of vehicle, 100.0 mg/kg 4OH-AB-PINACA, or 10.0 mg/kg rimonabant prior to 100.0 mg/kg 4OH-AB-PINACA detects a significant difference among groups (**Figure 5B**, inset; *F* = 16.951, *P* < 0.05). Each treatment group included five mice that were experimentally naive prior to initiation of these studies. Importantly, this difference is driven by 4OH-AB-PINACA alone eliciting a hypothermic effect which is significantly different from that observed following injection of the vehicle (*q* = 8.234, *P* < 0.05) and from that observed following administration of rimonabant with AB-PINACA (*q* = 4.271, *P* < 0.05). Rimonabant does not alter rectal temperature at the 10 mg/kg dose used in these studies. Similar to the parent drug, these data demonstrate dose-dependent hypothermic effects of 4OH-AB-PINACA which are mediated by agonist actions at CB1Rs in the mouse. The doses selected for these *in vivo* studies were based on preliminary experiments with AB-PINACA and our previous work with hydroxylated metabolites of indole-alkylamine-derived SCBs JWH-018 (Brents et al., 2011) and JWH-073 (Brents et al., 2012b), which demonstrated that the metabolites are typically ∼10-fold less potent than the parents after systemic injection. We present here a range of doses of AB-PINACA beginning with a small dose that is essentially devoid of hypothermic effects, then proceed in half-log units to a dose which elicits a robust hypothermic response. Because quantities of 4OH-AB-PINACA were limited, we tested only doses 10-fold higher than those that were active for the parent drug.

## Discussion

These studies demonstrate that when compared to Δ^9^-THC, the popular SCB AB-PINACA exhibits similar hCB1R affinity, but greater efficacy for G-protein activation and higher potency for adenylyl cyclase inhibition. Furthermore, chronic exposure of cells to AB-PINACA results in greater desensitization of CB1Rs than Δ^9^-THC. In humans, AB-PINACA is metabolized to two major phase one metabolites (4OH- and 5OH-AB-PINACA) that importantly retain high nM affinity and full agonist activity at CB1Rs. 4OH-AB-PINACA and 5OH-AB-PINACA are also poorly glucuronidated *in vitro* when compared to JWH-018-M2, a major monohydroxylated metabolite of the first generation SCB JWH-018. Finally, AB-PINACA and 4OH-AB-PINACA are active *in vivo*, producing CB1R-mediated hypothermia in mice. Taken collectively, the atypical pharmacodynamic properties of AB-PINACA at CB1Rs relative to Δ^9^-THC (e.g., higher potency/efficacy and greater production of desensitization), coupled with an unusual metabolic profile (e.g., production of metabolically stable active phase I metabolites) may contribute to the pronounced adverse effects observed with abuse of this SCB compared to marijuana.

Similar to previously published reports (Wohlfarth et al., 2015), the present results demonstrate that humans metabolize AB-PINACA to the urinary 4OH- and 5OH- metabolites. This is consistent with previous reports describing the human metabolic pathways of other similar SCBs (Fantegrossi et al., 2014; Ford et al., 2017b). Often not examined and/or reported in clinical cases, no other confounding drugs or medications of the over 200 tested were reported or detected in clinical specimens. However, other SCBs and SCB metabolites were detected in this patient along with AB-PINACA, therefore complicating the clinical picture of SCB toxicity as a class. Indeed, most seized K2/Spice products contain from 3 to as many as 5 different SCBs (Seely et al., 2012, 2013). Animal studies show that co-administration of individual SCBs such as JWH-073 and JWH-018 (Brents et al., 2013), or JWH-073 and JWH-250 (Ossato et al., 2016) results in a synergistic enhancement of not only potentially beneficial, but also adverse effects of these compounds. In marked contrast to K2/Spice products, marijuana contains only a single psychoactive compound Δ^9^-THC and a second natural constituent known as cannabidiol, that appears to blunt adverse effects produced by Δ^9^-THC (Valdeolivas et al., 2012; Todd and Arnold, 2016). In fact, the beneficial combination of cannabidiol with Δ^9^-THC led to development of Sativex, a drug currently in clinical trials to treat a variety of indications including spasticity associated with multiple sclerosis (Keating, 2017). In addition to Δ^9^-THC and cannabidiol, the cannabis plant contains hundreds of other phytocannabinoids and constituents not present in K2/Spice products that may help mitigate harmful and/or adverse effects (Prandi et al., 2018).

In addition to potential harmful synergistic effects of multiple SCBs in K2/Spice products, *in vitro* data presented here indicate that AB-PINACA is as full agonist that binds with high affinity for CB1Rs, in contrast to Δ^9^-THC that acts as a partial CB1 agonist (Bonhaus et al., 1998; Canazza et al., 2016). The full hCB1 agonist activity of AB-PINACA reported here is not only consistent with a previous report for this SCB (Costain et al., 2016), but is also similar to full efficacy of many other SCBs present in K2/Spice products (e.g., JWH-018, 5F-PB-22, MAM-2201, JWH-250, STS-135, and XLR-11) to activate G-proteins and to inhibit adenylyl cyclase activity (Brents et al., 2011; Wiley et al., 2012, 2013; Costain et al., 2016; De Luca et al., 2016). The high affinity and greater activity of AB-PINACA and other commonly abused SCBs at CB1Rs relative to Δ^9^-THC might be expected to produce enhanced psychotropic and adverse effects, contributing to the reported abuse and marked toxicity of this novel SCB.

Consistent with enhanced acute effects relative to Δ^9^-THC, chronic exposure to the full agonist AB-PINACA also resulted in greater desensitization/tolerance of CB1Rs the partial agonist Δ^9^-THC. Several reports have documented that a significant population of K2/Spice users smoke both marijuana and products containing SCBs on a daily basis (Cooper, 2016) and for extended periods of time (Hu et al., 2011; Vandrey et al., 2012). Chronic cannabinoid administration produces reduced effects when examined in cellular models by employing *in vitro* methods (Pertwee, 2005) and in animals by using in *vivo* approaches (Dewey, 1986; Abood and Martin, 1992; Maldonado and Rodríguez de Fonseca, 2002). Prolonged marijuana use in humans also results in decreased drug effects, indicative of tolerance (Jones et al., 1981; Hollister, 1986; Ramaekers et al., 2011). Since in this study chronic administration of the full agonist AB-PINACA produced greater cellular desensitization of CB1Rs than Δ^9^-THC, it is also probable that prolonged use of this SCB results in greater tolerance, potentially leading to dependence, in humans. Indeed, significant tolerance (Every-Palmer, 2011) and withdrawal has been observed in patients undergoing detoxification from SCB use in a report from New Zealand (Macfarlane and Christie, 2015). Perhaps more importantly, due to cross tolerance, it might be expected that users with a history of Δ^9^-THC use would increase SCBs abuse to produce desired psychoactive effects relative to naive users. Higher SCB doses in this population might result in interaction with non-cannabinoid off-target sites possibly leading to increased toxicity.

Results reported here also suggest that atypical metabolism may contribute to toxicity of AB-PINACA. For example, in marked contrast to metabolism of Δ^9^-THC that leads to a only a single phase I hydroxylated metabolite (Compton et al., 1993), AB-PINACA undergoes various types of metabolism, mostly oxidation and hydrolysis reactions, to form more than 20 different metabolites in human liver (Wohlfarth et al., 2015). Among these, 4OH-AB-PINACA and 5OH-AB-PINACA are the major oxidized metabolites of AB-PINACA. In addition to oxidation and hydrolysis, glucuronidation is an important metabolic pathway in the biotransformation of xenobiotics. In this study, we tested whether glucuronidation was involved in the hepatic clearance of 4OH- and 5OH-AB-PINACA. Although glucuronidation is not involved in the clearance of 5OH-AB-PINACA, 4OH-AB-PINACA is slightly glucuronidated in HLMs (**Figure 4C**). However, further investigation demonstrated that glucuronidation of 4OH-AB-PINACA is much less than that of JWH-018-M2 (**Figure 4D**), one of the major oxidized metabolites of JWH-018 (Brents et al., 2011, 2012b). Our observations therefore indicate that 4OH-AB-PINACA and 5OH-AB-PINACA are metabolically stable in the liver. Thus, accumulation of active phase I metabolites of AB-PINACA might lead to additive, synergistic and/or extended psychotropic and toxic effects relative to Δ^9^-THC. Potential participation of active metabolites in SCB toxicity as suggested by the present study indicates an increasing need for development of sensitive methods for quick and cost-effective detection of these metabolites in biological fluids. Such techniques are being developed and a very recent study demonstrated the use of ultra-high pressure liquid chromatography quadrupole time of flight mass spectrometry (UHPLC-QTOF-MS) to identify and quantify urinary metabolites for a broad range of SCBs seized in Norway (Gundersen et al., 2018).

Like other CB1R agonists, AB-PINACA elicits dose-dependent hypothermic effects which are attenuated by pretreatment with the CB1R inverse agonist/antagonist rimonabant. This same pattern of results has been previously obtained in mice with phytocannabinoids like Δ^9^-THC (Compton et al., 1996), endocannabinoids like anandamide (Cravatt et al., 2001), and “first generation” SCB agonists like JWH-018 (Brents et al., 2011; De Luca et al., 2015) suggesting that all of these structurally diverse compounds elicit hypothermic effects in the mouse *via* agonist actions at CB1Rs. We have previously reported that phase one hydroxylated metabolites of JWH-018 and JWH-073 (Brents et al., 2012b) also retain biological activity, with those exhibiting agonist actions *in vitro* also eliciting hypothermic effects in the mouse. Here we extend those findings from the indole-based SCBs to the indazole-based SCB AB-PINACA and its 4OH- metabolite. It is interesting to note that approximately a 10-fold greater dose of the metabolite 4OH-AB-PINACA (100 mg/kg) is required to produce a similar level of hypothermia when compared to the parent compound AB-PINACA (10 mg/kg). This is likely reflected by results from *in vitro* studies, in which it was found that the 4OH metabolite exhibits an affinity for hCB1Rs that is approximately 40-fold less (159 nM) than that of the parent compound (4 nM). In addition to reduced hCB1R affinity, physiochemical properties of the metabolite would also be expected to result in reduced entry into the brain because of the introduction of a polar hydroxyl group at the 4-position. Both of these factors would be expected to decrease *in vivo* potency for elicitation of centrally mediated effects, as those observed in the present study.

## Conclusion

Data reported here suggest that atypical pharmacodynamic properties of AB-PINACA at CB1Rs relative to Δ^9^-THC (e.g., higher potency/efficacy and greater production of desensitization), coupled with an unusual metabolic profile (e.g., production of metabolically stable active phase I metabolites) may contribute to the pronounced adverse effects observed with abuse of this SCB compared to marijuana.

## Author Contributions

MS, LJ, JM, WF, AP, PP, and DF participated in research design. RH, BF, LF, CW, AY, RF, AP, and DF conducted the experiments. MS, LJ, JM, WF, and AP contributed new reagents or analytic tools. RF, LJ, JM, WF, AR-P, PP, AP, and DF performed the data analysis. RF, LJ, JM, WF, AR-P, PP, and AP wrote or contributed to the writing of the manuscript.

## Conflict of Interest Statement

JM and AP were employed by the company PinPoint Testing, LLC. The remaining authors declare that the research was conducted in the absence of any commercial or financial relationships that could be construed as a potential conflict of interest.

## Abbreviations

Δ^9^-THC: Δ^9^-tetrahydrocannabinol
AB-PINACA: [(S)-N1(1-amino-3-methyl-1-oxobutan-2-yl)-1-pentyl-1H-indazole-3-carboxamide]
CB1R: cannabinoid receptor type 1
CB2R: cannabinoid receptor type 2
CBR: cannabinoid receptor
CDDR: Center for Drug Detection and Response
CHO-hCB1: Chinese hamster ovary cells expressing human CB1 receptors
CYP: specific P450
HLMs: human liver microsomes
LC-MS/MS: liquid chromatography-tandem mass spectrometry
NPSs: novel psychoactive substances
P450: cytochrome P450
SCB: synthetic cannabinoid
UGTs: uridine diphosphate (UDP)-glucuronosyltransferases
UPLC: ultra-performance liquid chromatography
