# Supplementary material for: Atypical Pharmacodynamic Properties and Metabolic Profile of the Abused Synthetic Cannabinoid AB-PINACA: Potential Contribution to Pronounced Adverse Effects Relative to Δ9-THC
Source: Front Pharmacol. 2018 Sep 26;9:1084. doi: 10.3389/fphar.2018.01084 (PMC6168621; doi:10.3389/fphar.2018.01084)
Supplement: Supplementary file 1 [file Table_1.DOCX]

Supplementary Material

**Atypical Pharmacodynamic Properties and Metabolic Profile of the Abused Synthetic Cannabinoid AB-PINACA: Potential Contribution to Pronounced Adverse Effects Relative to ∆^9^-THC**

**Rachel D. Hutchison^1^, Benjamin M. Ford^1^ Lirit N. Franks^1^, Catheryn D. Wilson^1^, Azure L. Yarbrough^2^, Ryoichi Fujiwara^2^, Mark K. Su^3^, Denise Fernandez^4^, Laura P. James^5^, Jeffery H. Moran^6^, Amy L. Patton^6^, William E. Fantegrossi^1^, Anna Radominska-Pandya^2^ and Paul L. Prather^1*^**

*** Correspondence:** Paul L Prather: pratherpaull@uams.edu

# **Supplementary Tables**

**Supplemental Table 1.** List of analytes

| 25C-NBOMe | Clomipramine | Morphine |
| --- | --- | --- |
| 25I-NBOMe | Clonazepam | Naloxone |
| 2C-B | Clozapine | Naltrexol |
| 2-Hydroxyethylflurazepam | Cocaine | Naltrexone |
| 4-APB | Codeine | Naphyrone |
| 4-BMC | Cotinine | Nitrazepam |
| 4-Ethylethcathinone | Cyclobenzaprine | NM2201 |
| 4-Ethylmethcathinone | Desalkylflurazepam | Norbuprenorphine |
| 4-Methylethcathinone | Desipramine | Nordiazepam |
| 5-F-AB-PINACA | Desmethylclobazam | Norephedrine |
| 5-F-ADBICA | Desmethylclomipramine | Norfentanyl |
| 5-F-ADB-PINACA | Desmethyldoxepin | Norfluoxetine |
| 5-F-APINACA | Desmethylmirtazapine | Norhydrocodone |
| 5-fluoro ADB metabolite 7 | Desmethyltapentadol | Norketamine |
| 5-fluoro ADB | Desmethyltrimipramine | Normeperidine |
| 5-fluoro-PB-22-3-carboxyindole | Dextromethorphan | Noroxycodone |
| 5-F-PB-22 | Dextrorphan | Noroxymorphone |
| 5-F-PINACA | Diazepam | Norpropoxyphene |
| 5-MAPB | Dihydrocodeine | Norsertraline |
| 5-MeO-DiPT | Diphenhydramine | Nortriptyline |
| 5-MeO-DMT | DMT | O-desmethyl-tramadol |
| 5-MeO-MiPT | Doxepin | O-Desmethylvenlafaxine |
| 6-APDB | Doxylamine | Ondansetron |
| 6-MAM | Duloxetine | Oxazepam |
| 7-aminoclonazepam | EDDP | Oxycodone |
| 7-aminoflunitrazepam | Estazolam | Oxymorphone |
| 7-Aminonitrazepam | Ethylone | Paroxetine |
| 7-APDB | Ethylphenidate | PB-22 |
| 7-OH-quetiapine | Etizolam | Pentazocine |
| AB-CHMINACA M1A | Fentanyl | Pentedrone |
| AB-CHMINACA M3A | Flunitrazepam | Pentylone |
| AB-CHMINACA | Fluoxetine | Phenazepam |
| AB-FUBINACA | Fluphenazine | Phencyclidine |
| AB-PINACA | Flurazepam | Phentermine |
| AB-PINACA-(5-hydroxypentyl) | FUB-AKB-48 | Phenytoin |
| Acetaminophen | FUBIMINA | PMMA |
| Acetyl Fentanyl | FUB-PB-22 | Prazepam |
| Acetyl Norfentanyl | Haloperidol | Primidone |
| ADB-FUBINACA | Hydrocodone | Promethazine |
| ADBICA | Hydromorphone | Propoxyphene |
| ADB-PINACA | Imipramine | Protriptyline |
| a-hydroxyalprazolam | JWH-018 | Pseudoephedrine |
| a-hydroxymidazolam | JWH-018-pentanoic acid | PV8 |
| a-hydroxytriazolam | Ketamine | Quetiapine |
| Alfentanil | Levamisole | Risperidone |
| alpha-PPP | Lorazepam | Sertraline |
| alpha-PVP | MAB-CHMINACA M1 | Sufentanil |
| Alprazolam | MAB-CHMINACA M10 | Tapentadol |
| AM2201 | MAB-CHMINACA M11 | Temazepam |
| Amiodarone | MAB-CHMINACA M3 | TFMPP |
| Amitriptyline | MAB-CHMINACA | THJ018 |
| Amoxapine | Maprotiline | Topiramate |
| Amphetamine | MBZP | Tramadol |
| APICA | mCPP | Trazodone |
| APINACA (aka AKB-48) | MDA | Triazolam |
| Aripiprazole | MDAI | Trimipramine |
| Atropine | MDEA | UR-144 Degradant |
| Benzoylecgonine | MDMA | UR-144 |
| Buprenorphine | MDMB-CHMINACA | UR-144-(5-hydroxypentyl) |
| Bupropion | MDPV | UR-144-pentanoic acid |
| Buspirone | Meperidine | UR-144-pentanoic acid degradant |
| Butylone | Meprobamate | Venlafaxine |
| BZP | Methadone | Verapamil |
| Caffeine | Methamphetamine | XLR11 Degradant |
| Carbamazepine | Methylone | XLR-11 |
| Carisoprodol | Methylphenidate | XLR11-(4-hydroxypentyl) |
| Chlordiazepoxide | Metoprolol | Zaleplon |
| Chlorpromazine | Midazolam | Zolpidem |
| Citalopram | Mirtazapine | Zopiclone |

**Supplemental Table 2.** Instrument configuration for Agilent 6420.

| **Parameter** | **Setting** |
| --- | --- |
| Mass spectrometer mode | Positive electrospray ionization, dynamic multiple reaction monitoring (dMRM) |
| Gas Temperature | 350 ºC |
| Gas Flow | 12 L/min [Nitrogen] |
| Nebulizer Gas | 50 psi [Nitrogen] |
| Capillary Voltage | 3500 V |
| Cell Accelerator Voltage | 4 V |

**Supplemental Table 3.** Mass spectrometry parameters for Specific Reaction Monitoring (SRM)

experiments for Agilent 6420.

| MS Method | Precursor | Product 1 | Product 2 | Analyte | Fragmentor (V) | Collision Energy (V) - Product 1 | Collision Energy (V) -Product 2 |
| --- | --- | --- | --- | --- | --- | --- | --- |
| PQ 1 | 333.1 | 211 | 51.3 | 2-Hydroxyethylflurazepam | 148 | 41 | 145 |
| PQ 1 | 337.1 | 215 | - | 2-Hydroxyethylflurazepam-D4 | 143 | 45 | - |
| PQ 1 | 328.2 | 211 | 165 | 6-MAM | 164 | 25 | 45 |
| PQ 1 | 334.2 | 165 | - | 6-MAM-D6 | 188 | 53 | - |
| PQ 1 | 286.1 | 222 | 121 | 7-Aminoclonazepam | 158 | 25 | 33 |
| PQ 1 | 290.1 | 121 | - | 7-Aminoclonazepam-D4 | 168 | 33 | - |
| PQ 1 | 252.1 | 121.1 | 94.2 | 7-Aminonitrazepam | 138 | 29 | 45 |
| PQ 1 | 257.1 | 121.1 | - | 7-Aminonitrazepam-D5 | 153 | 29 | - |
| PQ 1 | 323.2 | 188 | 105.1 | Acetyl Fentanyl | 152 | 21 | 45 |
| PQ 1 | 328.2 | 105.1 | - | Acetyl Fentanyl-D5 | 134 | 45 | - |
| PQ 1 | 219.1 | 84.2 | 55.3 | Acetyl Norfentanyl | 80 | 13 | 41 |
| PQ 1 | 224.2 | 84.2 | - | Acetyl Norfentanyl-D5 | 124 | 17 | - |
| PQ 1 | 325.1 | 297 | 215.9 | alpha-hydroxyalprazolam | 180 | 25 | 45 |
| PQ 1 | 309.1 | 281 | 205 | Alprazolam | 160 | 29 | 49 |
| PQ 1 | 314.1 | 286 | - | Alprazolam-D5 | 180 | 29 | - |
| PQ 1 | 136.1 | 119 | 91.1 | Amphetamine | 65 | 5 | 17 |
| PQ 1 | 147.2 | 130.1 | - | Amphetamine-D11 | 90 | 9 | - |
| PQ 1 | 290.1 | 168 | 105 | Benzoylecgonine | 126 | 17 | 29 |
| PQ 1 | 298.2 | 171 | - | Benzoylecgonine-D8 | 128 | 21 | - |
| PQ 1 | 468.3 | 414.2 | 101.1 | Buprenorphine | 212 | 41 | 53 |
| PQ 1 | 472.3 | 59.3 | - | Buprenorphine-D4 | 216 | 60 | - |
| PQ 1 | 261.2 | 176 | 55.3 | Carisoprodol | 92 | 5 | 33 |
| PQ 1 | 268.2 | 183.1 | - | Carisoprodol-D7 | 92 | 5 | - |
| PQ 1 | 316 | 269.9 | 213.8 | Clonazepam | 160 | 25 | 45 |
| PQ 1 | 304.2 | 182 | 105 | Cocaine | 132 | 17 | 37 |
| PQ 1 | 307.2 | 185 | - | Cocaine-D3 | 138 | 21 | - |
| PQ 1 | 300.2 | 215 | 165 | Codeine | 150 | 25 | 49 |
| PQ 1 | 306.2 | 218 | - | Codeine-D6 | 162 | 29 | - |
| PQ 1 | 276.2 | 231 | 214.9 | Cyclobenzaprine | 126 | 9 | 49 |
| PQ 1 | 279.2 | 215 | - | Cyclobenzaprine-D3 | 138 | 45 | - |
| PQ 1 | 272.2 | 171 | 147 | Dextromethorphan | 156 | 45 | 33 |
| PQ 1 | 275.2 | 171 | - | Dextromethorphan-D3 | 160 | 45 | - |
| PQ 1 | 258.2 | 157 | 133 | Dextrorphan | 168 | 45 | 33 |
| PQ 1 | 261.2 | 157 | - | Dextrorphan-D3 | 158 | 45 | - |
| PQ 1 | 285.1 | 193 | 153.9 | Diazepam | 154 | 33 | 29 |
| PQ 1 | 290.1 | 198 | - | Diazepam-D5 | 154 | 37 | - |
| PQ 1 | 302.2 | 198.9 | 128 | Dihydrocodeine | 152 | 33 | 60 |
| PQ 1 | 308.2 | 202 | - | Dihydrocodeine-D6 | 160 | 37 | - |
| PQ 1 | 278.2 | 250 | 235 | EDDP | 168 | 25 | 33 |
| PQ 1 | 282.2 | 235 | - | EDDP-D3 | 162 | 33 | - |
| PQ 1 | 337.2 | 188 | 105.1 | Fentanyl | 158 | 21 | 45 |
| PQ 1 | 342.3 | 105.1 | - | Fentanyl-D5 | 158 | 45 | - |
| PQ 1 | 314.1 | 268 | 239 | Flunitrazepam | 162 | 29 | 41 |
| PQ 1 | 388.2 | 317 | 315 | Flurazepam | 150 | 17 | 25 |
| PQ 1 | 300.2 | 198.9 | 128 | Hydrocodone | 168 | 33 | 60 |
| PQ 1 | 306.2 | 202 | - | Hydrocodone-D6 | 164 | 33 | - |
| PQ 1 | 286.1 | 184.9 | 157 | Hydromorphone | 162 | 33 | 49 |
| PQ 1 | 292.2 | 185 | - | Hydromorphone-D6 | 166 | 37 | - |
| PQ 1 | 238.1 | 220 | 125 | Ketamine | 104 | 13 | 29 |
| PQ 1 | 242.1 | 224 | - | Ketamine-D4 | 108 | 13 | - |
| PQ 1 | 321 | 303 | 274.9 | Lorazepam | 132 | 13 | 21 |
| PQ 1 | 180.1 | 163 | 105.1 | MDA | 90 | 9 | 25 |
| PQ 1 | 185.1 | 168 | - | MDA-D5 | 92 | 9 | - |
| PQ 1 | 208.1 | 163 | 105.1 | MDEA | 102 | 9 | 25 |
| PQ 1 | 214.2 | 166 | - | MDEA-D6 | 102 | 9 | - |
| PQ 1 | 194.1 | 163 | 105.1 | MDMA | 94 | 9 | 29 |
| PQ 1 | 200.2 | 166 | - | MDMA-D6 | 90 | 9 | - |
| PQ 1 | 248.2 | 220 | 174 | Meperidine | 138 | 21 | 17 |
| PQ 1 | 219.1 | 158 | 55.3 | Meprobamate | 92 | 5 | 25 |
| PQ 1 | 226.2 | 165.1 | - | Meprobamate-D7 | 92 | 5 | - |
| PQ 1 | 310.2 | 265 | 105.1 | Methadone | 120 | 29 | 13 |
| PQ 1 | 319.3 | 268.1 | - | Methadone-D9 | 126 | 13 | - |
| PQ 1 | 150.1 | 119 | 91.1 | Methamphetamine | 96 | 9 | 21 |
| PQ 1 | 161.2 | 97.1 | - | Methamphetamine-D11 | 94 | 21 | - |
| PQ 1 | 326.1 | 249 | 223 | Midazolam | 184 | 41 | 45 |
| PQ 1 | 330.1 | 295.1 | - | Midazolam-D4 | 196 | 29 | - |
| PQ 1 | 286.2 | 164.8 | 152.5 | Morphine | 164 | 49 | 53 |
| PQ 1 | 292.2 | 152.6 | - | Morphine-D6 | 164 | 53 | - |
| PQ 1 | 328.2 | 309.8 | 211.8 | Naloxone | 132 | 17 | 45 |
| PQ 1 | 333.2 | 333.2 | - | Naloxone-D5 | 134 | 21 | - |
| PQ 1 | 344.2 | 326.1 | 308.1 | Naltrexol | 136 | 21 | 29 |
| PQ 1 | 347.2 | 329.1 | - | Naltrexol-D3 | 138 | 25 | - |
| PQ 1 | 342.2 | 324.1 | 55.3 | Naltrexone | 122 | 21 | 45 |
| PQ 1 | 282.1 | 236 | 180 | Nitrazepam | 156 | 25 | 41 |
| PQ 1 | 414.3 | 101.1 | 57.3 | Norbuprenorphine | 216 | 41 | 49 |
| PQ 1 | 417.3 | 101.1 | - | Norbuprenorphine-D3 | 226 | 41 | - |
| PQ 1 | 271.1 | 208 | 140 | Nordiazepam | 160 | 29 | 29 |
| PQ 1 | 276.1 | 140 | - | Nordiazepam-D5 | 164 | 33 | - |
| PQ 1 | 233.2 | 84.2 | 55.3 | Norfentanyl | 102 | 17 | 41 |
| PQ 1 | 238.2 | 84.2 | - | Norfentanyl-D5 | 106 | 17 | - |
| PQ 1 | 286.2 | 199 | 171 | Norhydrocodone | 218 | 29 | 45 |
| PQ 1 | 289.2 | 202 | - | Norhydrocodone-D3 | 184 | 29 | - |
| PQ 1 | 234.1 | 160 | 42.3 | Normeperidine | 122 | 13 | 41 |
| PQ 1 | 238.2 | 164 | - | Normeperidine-D4 | 130 | 13 | - |
| PQ 1 | 302.1 | 284 | 186.9 | Noroxycodone | 126 | 13 | 25 |
| PQ 1 | 305.2 | 287 | - | Noroxycodone-D3 | 138 | 13 | - |
| PQ 1 | 326.2 | 252.1 | 44.3 | Norpropoxyphene | 94 | 13 | 0 |
| PQ 1 | 331.2 | 44.3 | - | Norpropoxyphene-D5 | 98 | 9 | - |
| PQ 1 | 250.2 | 58.3 | 42.3 | O-desmethyl-tramadol | 96 | 13 | 60 |
| PQ 1 | 256.2 | 64.3 | - | O-desmethyl-tramadol-D6 | 94 | 17 | - |
| PQ 1 | 287.1 | 268.9 | 240.9 | Oxazepam | 136 | 13 | 21 |
| PQ 1 | 292.1 | 246 | - | Oxazepam-D5 | 132 | 21 | - |
| PQ 1 | 316.2 | 298 | 241 | Oxycodone | 126 | 17 | 29 |
| PQ 1 | 322.2 | 304.1 | - | Oxycodone-D6 | 134 | 17 | - |
| PQ 1 | 302.1 | 284 | 227 | Oxymorphone | 150 | 17 | 29 |
| PQ 1 | 305.2 | 287 | - | Oxymorphone-D3 | 158 | 17 | - |
| PQ 1 | 351 | 206 | 183.8 | Phenazepam | 186 | 41 | 33 |
| PQ 1 | 355 | 183.9 | - | Phenazepam-D4 | 180 | 41 | - |
| PQ 1 | 244.2 | 91.1 | 86.2 | Phencyclidine | 90 | 37 | 9 |
| PQ 1 | 249.2 | 86.2 | - | Phencydlidine-D5 | 92 | 9 | - |
| PQ 1 | 150.1 | 92.1 | 65.2 | Phentermine | 71 | 17 | 49 |
| PQ 1 | 155.2 | 96.2 | - | Phentermine-D5 | 71 | 21 | - |
| PQ 1 | 340.2 | 266.1 | 58.3 | Propoxyphene | 92 | 4 | 13 |
| PQ 1 | 351.3 | 277.2 | - | Propoxyphene-D11 | 98 | 5 | - |
| PQ 1 | 387.2 | 238 | 111 | Sufentanil | 136 | 17 | 45 |
| PQ 1 | 392.2 | 238 | - | Sufentanil-D5 | 128 | 17 | - |
| PQ 1 | 222.2 | 121 | 107 | Tapentadol | 130 | 17 | 25 |
| PQ 1 | 225.2 | 107 | - | Tapentadol-D3 | 132 | 25 | - |
| PQ 1 | 301.1 | 283 | 255 | Temazepam | 132 | 9 | 25 |
| PQ 1 | 306.1 | 260 | - | Temazepam-D5 | 128 | 21 | - |
| PQ 1 | 264.2 | 58.3 | 43.3 | Tramadol | 98 | 13 | 60 |
| PQ 1 | 268.2 | 58.3 | - | Tramadol-13C-D3 | 102 | 17 | - |
| PQ 2 | 284.1 | 135.1 | 93.2 | 7-Aminoflunitrazepam | 153 | 65 | 33 |
| PQ 2 | 291.2 | 138.1 | - | 7-Aminoflunitrazepam-D7 | 140 | 33 | - |
| PQ 2 | 400.2 | 269 | 208 | 7-OH-quetiapine | 165 | 25 | 53 |
| PQ 2 | 152.1 | 43.3 | 110.1 | Acetaminophen | 89 | 13 | 37 |
| PQ 2 | 342.1 | 324 | 203 | alpha-hydroxymidazolam | 145 | 21 | 29 |
| PQ 2 | 346.1 | 328.1 | - | alpha-hydroxymidazolam-D4 | 140 | 21 | - |
| PQ 2 | 359.1 | 331 | 176 | alpha-hydroxytriazolam | 157 | 29 | 29 |
| PQ 2 | 646 | 100.2 | 58.3 | Amiodarone | 202 | 37 | 65 |
| PQ 2 | 650.1 | 58.3 | - | Amiodarone-D4 | 207 | 65 | - |
| PQ 2 | 314.1 | 271 | 193 | Amoxapine | 160 | 25 | 49 |
| PQ 2 | 240.1 | 184 | 131 | Bupropion | 84 | 9 | 29 |
| PQ 2 | 249.2 | 185 | - | Bupropion-D9 | 99 | 9 | - |
| PQ 2 | 195.1 | 138.1 | 42.3 | Caffeine | 120 | 21 | 53 |
| PQ 2 | 198.1 | 140 | - | Caffeine-13C3 | 112 | 21 | - |
| PQ 2 | 325.2 | 262 | 109.1 | Citalopram | 136 | 17 | 29 |
| PQ 2 | 331.2 | 109.1 | - | Citalopram-D6 | 135 | 25 | - |
| PQ 2 | 177.1 | 98.1 | 80.2 | Cotinine | 124 | 21 | 25 |
| PQ 2 | 180.1 | 101.1 | - | Cotinine-D3 | 124 | 21 | - |
| PQ 2 | 289.1 | 226 | 140 | Desalkylflurazepam | 108 | 29 | 33 |
| PQ 2 | 293.1 | 140 | - | Desalkylflurazepam-D4 | 155 | 33 | - |
| PQ 2 | 267.2 | 72.3 | 44.3 | Desipramine | 108 | 13 | 45 |
| PQ 2 | 270.2 | 75.3 | - | Desipramine-D3 | 95 | 13 | - |
| PQ 2 | 287.1 | 245 | 210 | Desmethylclobazam | 128 | 17 | 33 |
| PQ 2 | 293.1 | 251 | - | Desmethylclobazam-13C6 | 110 | 17 | - |
| PQ 2 | 252.2 | 195 | 209 | Desmethylmirtazapine | 140 | 21 | 21 |
| PQ 2 | 208.2 | 121.1 | 107.1 | Desmethyltapentadol | 116 | 17 | 25 |
| PQ 2 | 256.2 | 167 | 152 | Diphenhydramine | 89 | 9 | 45 |
| PQ 2 | 259.2 | 167 | - | Diphenhydramine-D3 | 74 | 13 | - |
| PQ 2 | 271.2 | 182 | 167 | Doxylamine | 94 | 13 | 41 |
| PQ 2 | 276.2 | 187.1 | - | Doxylamine-D5 | 99 | 13 | - |
| PQ 2 | 295.1 | 267 | 205 | Estazolam | 145 | 25 | 49 |
| PQ 2 | 300.1 | 272 | - | Estazolam-D5 | 135 | 25 | - |
| PQ 2 | 343.1 | 314 | 258.9 | Etizolam | 160 | 25 | 37 |
| PQ 2 | 281.2 | 86.2 | 58.3 | Imipramine | 120 | 17 | 49 |
| PQ 2 | 284.2 | 61.3 | - | Imipramine-D3 | 120 | 17 | - |
| PQ 2 | 205.1 | 117.1 | 91.2 | Levamisole | 135 | 25 | 53 |
| PQ 2 | 205.1 | 178 | 91.2 | Maprotiline | 118 | 21 | 45 |
| PQ 2 | 234.2 | 84.2 | 56.3 | Methylphenidate | 112 | 21 | 57 |
| PQ 2 | 243.2 | 93.2 | - | Methylphenidate-D9 | 116 | 21 | - |
| PQ 2 | 268.2 | 116.1 | 56.3 | Metoprolol | 133 | 17 | 29 |
| PQ 2 | 152.1 | 134.1 | 117.1 | Norephedrine | 70 | 9 | 17 |
| PQ 2 | 155.1 | 137.1 | - | Norephedrine-D3 | 70 | 9 | - |
| PQ 2 | 224.1 | 207 | 125 | Norketamine | 91 | 9 | 25 |
| PQ 2 | 228.1 | 129 | - | Norketamine-D4 | 87 | 25 | - |
| PQ 2 | 264.2 | 233 | 91.2 | Nortriptyline | 112 | 13 | 25 |
| PQ 2 | 267.2 | 233 | - | Nortriptyline-D3 | 120 | 9 | - |
| PQ 2 | 294.2 | 212 | 184.1 | Ondansetron | 133 | 17 | 25 |
| PQ 2 | 286.2 | 218.1 | 41.3 | Pentazocine | 136 | 17 | 61 |
| PQ 2 | 289.2 | 72.3 | - | Pentazocine-13C3 | 132 | 25 | - |
| PQ 2 | 253.1 | 182 | 104.1 | Phenytoin | 99 | 13 | 33 |
| PQ 2 | 263.2 | 192.1 | - | Phenytoin-D10 | 104 | 17 | - |
| PQ 2 | 325.1 | 271 | 140 | Prazepam | 136 | 25 | 45 |
| PQ 2 | 330.1 | 276 | - | Prazepam-D5 | 150 | 25 | - |
| PQ 2 | 219.1 | 162 | 91.2 | Primidone | 87 | 9 | 33 |
| PQ 2 | 285.1 | 86.2 | 71.3 | Promethazine | 94 | 17 | 53 |
| PQ 2 | 288.2 | 89.2 | - | Promethazine-D3 | 94 | 13 | - |
| PQ 2 | 264.2 | 161 | 155 | Protriptyline | 136 | 25 | 21 |
| PQ 2 | 267.2 | 155 | - | Protriptyline-D3 | 115 | 21 | - |
| PQ 2 | 166.1 | 148.1 | 117.1 | Pseudoephedrine | 70 | 9 | 21 |
| PQ 2 | 169.1 | 151.1 | - | Pseudoephedrine-D3 | 70 | 9 | - |
| PQ 2 | 384.2 | 253 | 221 | Quetiapine | 153 | 21 | 45 |
| PQ 2 | 392.2 | 226 | - | Quetiapine-D8 | 165 | 45 | - |
| PQ 2 | 372.2 | 176 | 148 | Trazodone | 153 | 25 | 41 |
| PQ 2 | 378.2 | 182 | - | Trazodone-D6 | 157 | 25 | - |
| PQ 2 | 278.2 | 260.2 | 58.3 | Venlafaxine | 109 | 9 | 17 |
| PQ 2 | 284.2 | 64.3 | - | Venlafaxine-D6 | 104 | 25 | - |
| PQ 2 | 455.3 | 165 | 150 | Verapamil | 163 | 29 | 49 |
| PQ 2 | 306.1 | 264 | 236 | Zaleplon | 136 | 21 | 29 |
| PQ 2 | 310.2 | 240 | - | Zaleplon-D4 | 161 | 29 | - |
| PQ 2 | 308.2 | 263 | 235 | Zolpidem | 157 | 25 | 41 |
| PQ 2 | 314.2 | 235.1 | - | Zolpidem-D6 | 155 | 37 | - |
| PQ 2 | 389.1 | 244.9 | 216.9 | Zopiclone | 91 | 13 | 37 |
| PQ 2 | 393.1 | 244.9 | - | Zopiclone-D4 | 91 | 17 | - |
| PQ 3 | 417.26 | 268.1 | 197.1 | Alfentanil | 121 | 13 | 25 |
| PQ 3 | 278.2 | 105.1 | 91.2 | Amitriptyline | 116 | 25 | 25 |
| PQ 3 | 281.2 | 91.2 | - | Amitriptyline-D3 | 120 | 25 | - |
| PQ 3 | 448.16 | 98.1 | 285 | Aripiprazole | 155 | 41 | 25 |
| PQ 3 | 456.21 | 293.1 | - | Aripiprazole-D8 | 160 | 25 | - |
| PQ 3 | 290.2 | 91.1 | 77.2 | Atropine | 138 | 49 | 73 |
| PQ 3 | 293.2 | 127.1 | - | Atropine-D3 | 133 | 21 | - |
| PQ 3 | 386.3 | 122 | 95.1 | Busiprone | 165 | 33 | 65 |
| PQ 3 | 394.3 | 122 | - | Busiprone-D8 | 160 | 41 | - |
| PQ 3 | 237.1 | 194 | 193.4 | Carbamazepine | 123 | 17 | 29 |
| PQ 3 | 243.1 | 200.1 | - | Carbamazepine-13C6 | 99 | 17 | - |
| PQ 3 | 300.1 | 282.7 | 227 | Chlordiazepoxide | 114 | 13 | 21 |
| PQ 3 | 305.1 | 286.1 | - | Chlordiazepoxide-D5 | 114 | 25 | - |
| PQ 3 | 319.11 | 58.2 | 86.2 | Chlorpromazine | 122 | 49 | 17 |
| PQ 3 | 322.12 | 89.2 | - | Chlorpromazine-D3 | 126 | 17 | - |
| PQ 3 | 315.2 | 58.3 | 86.2 | Clomipramine | 120 | 53 | 17 |
| PQ 3 | 318.2 | 89.2 | - | Clomipramine-D3 | 115 | 17 | - |
| PQ 3 | 327.14 | 270 | 192 | Clozapine | 121 | 21 | 53 |
| PQ 3 | 331.16 | 272 | - | Clozapine-D4 | 140 | 25 | - |
| PQ 3 | 301.2 | 72.3 | 44.3 | Desmethylclomipramine | 108 | 17 | 53 |
| PQ 3 | 304.2 | 75.3 | - | Desmethylclomipramine-D3 | 115 | 13 | - |
| PQ 3 | 266.2 | 107.1 | 44.3 | Desmethyldoxepin | 116 | 25 | 17 |
| PQ 3 | 269.2 | 107.1 | - | Desmethyldoxepin-D3 | 120 | 21 | - |
| PQ 3 | 299.14 | 198 | 256 | Desmethylolanzapine | 145 | 41 | 21 |
| PQ 3 | 307.19 | 198 | - | Desmethylolanzapine-D8 | 155 | 41 | - |
| PQ 3 | 281.2 | 44.3 | 86.2 | Desmethyltrimipramine | 110 | 41 | 13 |
| PQ 3 | 284.2 | 47.3 | - | Desmethyltrimipramine-D3 | 110 | 13 | - |
| PQ 3 | 264.2 | 58.2 | 246.1 | Desmethylvenlafaxine | 102 | 17 | 9 |
| PQ 3 | 270.24 | 64.2 | - | Desmethylvenlafaxine-D6 | 97 | 21 | - |
| PQ 3 | 280.2 | 107.1 | 77.2 | Doxepin | 128 | 25 | 65 |
| PQ 3 | 283.2 | 107.1 | - | Doxepin-D3 | 110 | 21 | - |
| PQ 3 | 298.13 | 44.2 | 154 | Duloxetine | 88 | 13 | 5 |
| PQ 3 | 301.15 | 47.2 | - | Duloxetine-D3 | 83 | 17 | - |
| PQ 3 | 310.1 | 44.3 | 148.1 | Fluoxetine | 95 | 5 | 13 |
| PQ 3 | 316.2 | 44.3 | - | Fluoxetine-D6 | 95 | 9 | - |
| PQ 3 | 438.18 | 171.1 | 143.1 | Fluphenazine | 146 | 25 | 33 |
| PQ 3 | 376.2 | 165 | 123 | Haloperidol | 138 | 25 | 45 |
| PQ 3 | 380.2 | 127 | - | Haloperidol-D5 | 138 | 49 | - |
| PQ 3 | 197.09 | 154 | 118 | mCPP | 107 | 21 | 41 |
| PQ 3 | 205.14 | 158 | - | mCPP-D8 | 116 | 21 | - |
| PQ 3 | 296.1 | 30.4 | 134.1 | Norfluoxetine | 70 | 5 | 5 |
| PQ 3 | 302.2 | 30.4 | - | Norfluoxetine-D6 | 70 | 5 | - |
| PQ 3 | 288.13 | 213 | 270.1 | Noroxymorphone | 140 | 29 | 13 |
| PQ 3 | 275 | 158.9 | 123 | Norsertraline | 111 | 17 | 49 |
| PQ 3 | 281 | 160 | - | Norsertraline-13C6 | 111 | 16 | - |
| PQ 3 | 330.15 | 70.2 | 192.1 | Paroxetine | 131 | 33 | 17 |
| PQ 3 | 336.19 | 76.2 | - | Paroxetine-D6 | 136 | 33 | - |
| PQ 3 | 411.22 | 191.1 | 69.1 | Risperidone | 146 | 29 | 69 |
| PQ 3 | 415.25 | 195.1 | - | Risperidone-D4 | 141 | 29 | - |
| PQ 3 | 306.1 | 275 | 158.9 | Sertraline | 79 | 9 | 25 |
| PQ 3 | 309.1 | 275 | - | Sertraline-D3 | 79 | 9 | - |
| PQ 3 | 340.11 | 264 | 184 | Topiramate | 126 | 5 | 5 |
| PQ 3 | 352.18 | 270 | - | Topiramate-D12 | 131 | 5 | - |
| PQ 3 | 343.05 | 308 | 314.9 | Triazolam | 170 | 25 | 29 |
| PQ 3 | 347.08 | 312 | - | Triazolam-D4 | 170 | 25 | - |
| PQ 3 | 295.2 | 58.3 | 100.2 | Trimipramine | 120 | 49 | 13 |
| PQ 3 | 298.2 | 103.2 | - | Trimipramine-D3 | 105 | 17 | - |
| PQ 4 | 336.1 | 121.1 | 91.2 | 25C-NBOMe | 104 | 17 | 49 |
| PQ 4 | 339.2 | 124.1 | - | 25C-NBOMe-D3 | 104 | 17 | - |
| PQ 4 | 428.1 | 121.1 | 91.2 | 25I-NBOMe | 118 | 21 | 65 |
| PQ 4 | 431.1 | 124.1 | - | 25I-NBOMe-D3 | 123 | 25 | - |
| PQ 4 | 260 | 242.9 | 227.9 | 2C-B | 89 | 9 | 21 |
| PQ 4 | 266.2 | 249 | - | 2C-B-D6 | 78 | 9 | - |
| PQ 4 | 176.1 | 159 | 131 | 4-APB | 74 | 9 | 17 |
| PQ 4 | 242 | 145 | 144.1 | 4-BMC | 94 | 13 | 37 |
| PQ 4 | 206.2 | 188.1 | 144.1 | 4-Ethylethcathinone | 94 | 9 | 33 |
| PQ 4 | 192.1 | 174.1 | 145.1 | 4-Ethylmethcathinone | 94 | 9 | 21 |
| PQ 4 | 192.1 | 174.1 | 145.1 | 4-Methylethcathinone | 99 | 9 | 21 |
| PQ 4 | 190.1 | 159 | 131 | 5-MAPB | 89 | 9 | 21 |
| PQ 4 | 275.2 | 174 | 114.1 | 5-MeO-DiPT | 99 | 21 | 13 |
| PQ 4 | 219.1 | 174 | 58.3 | 5-MeO-DMT | 89 | 13 | 13 |
| PQ 4 | 247.2 | 86.2 | 44.3 | 5-MeO-MiPT | 99 | 13 | 41 |
| PQ 4 | 178.1 | 161.1 | 133 | 6-APDB | 79 | 9 | 21 |
| PQ 4 | 204.1 | 105.1 | 98.2 | alpha-PPP | 118 | 25 | 25 |
| PQ 4 | 232.2 | 91.2 | 77.2 | alpha-PVP | 123 | 21 | 57 |
| PQ 4 | 240.2 | 91.2 | - | alpha-PVP-D8 | 133 | 25 | - |
| PQ 4 | 222.1 | 204 | 174.1 | Butylone | 99 | 9 | 17 |
| PQ 4 | 225.1 | 207 | - | Butylone-D3 | 104 | 9 | - |
| PQ 4 | 177.1 | 91.2 | 39.3 | BZP | 99 | 21 | 75 |
| PQ 4 | 184.2 | 98.2 | - | BZP-D7 | 99 | 21 | - |
| PQ 4 | 189.1 | 144.1 | 58.3 | DMT | 79 | 17 | 13 |
| PQ 4 | 222.1 | 204 | 174 | Ethylone | 94 | 9 | 17 |
| PQ 4 | 227.1 | 179.1 | - | Ethylone-D5 | 99 | 17 | - |
| PQ 4 | 248.2 | 84.2 | 56.3 | Ethylphenidate | 99 | 21 | 57 |
| PQ 4 | 191.2 | 91.2 | 65.2 | MBZP | 104 | 25 | 53 |
| PQ 4 | 178.1 | 161 | 103.1 | MDAI | 69 | 9 | 29 |
| PQ 4 | 276.2 | 135 | 126.1 | MDPV | 126 | 25 | 25 |
| PQ 4 | 284.2 | 175 | - | MDPV-D8 | 140 | 21 | - |
| PQ 4 | 208.1 | 160 | 132.1 | Methylone | 83 | 17 | 29 |
| PQ 4 | 211.1 | 163 | - | Methylone-D3 | 91 | 17 | - |
| PQ 4 | 282.2 | 211 | 141 | Naphyrone | 126 | 17 | 25 |
| PQ 4 | 287.2 | 141 | - | Naphyrone-D5 | 135 | 29 | - |
| PQ 4 | 192.1 | 174.1 | 91.2 | Pentedrone | 83 | 9 | 21 |
| PQ 4 | 236.1 | 218 | 188.1 | Pentylone | 104 | 9 | 13 |
| PQ 4 | 239.1 | 221.1 | - | Pentylone-D3 | 94 | 9 | - |
| PQ 4 | 180.1 | 149.1 | 121.1 | PMMA | 79 | 9 | 21 |
| PQ 4 | 183.2 | 149.1 | - | PMMA-D5 | 73 | 9 | - |
| PQ 4 | 260.2 | 91.2 | 77.2 | PV8 | 133 | 21 | 65 |
| PQ 4 | 231.1 | 188 | 44.3 | TFMPP | 113 | 21 | 25 |
| PQ 4 | 235.1 | 190 | - | TFMPP-D4 | 128 | 25 | - |
| PQ 4 | 362.2 | 232 | 345.2 | 5-Fluoro-ADBICA | 94 | 5 | 17 |
| PQ 4 | 349.2 | 332.2 | 233.1 | 5-Fluoro-AB-PINACA | 78 | 5 | 25 |
| PQ 4 | 363.2 | 233 | 346.2 | 5-Fluoro-ADB-PINACA | 89 | 5 | 25 |
| PQ 4 | 383.2 | 135.1 | 93.2 | 5-Fluoro-APICA | 143 | 29 | 65 |
| PQ 4 | 384.2 | 135.1 | 93.2 | 5-Fluoro-APINACA | 128 | 25 | 61 |
| PQ 4 | 377.2 | 232 | 144 | 5-Fluoro-PB-22 | 105 | 9 | 45 |
| PQ 4 | 357.2 | 312.2 | 241.1 | AB-CHMINACA | 89 | 13 | 25 |
| PQ 4 | 361.2 | 316.3 | - | AB-CHMINACA-D4 | 99 | 13 | - |
| PQ 4 | 369.2 | 253 | 109.1 | AB-FUBINACA | 95 | 25 | 49 |
| PQ 4 | 373.1 | 328.2 | - | AB-FUBINACA-D4 | 78 | 13 | - |
| PQ 4 | 331.2 | 314.2 | 215 | AB-PINACA | 85 | 5 | 21 |
| PQ 4 | 340.1 | 224.2 | - | AB-PINACA-D9 | 63 | 25 | - |
| PQ 4 | 383.2 | 109.1 | 338.2 | ADB-FUBINACA | 89 | 13 | 57 |
| PQ 4 | 344.2 | 214 | 327.2 | ADBICA | 94 | 5 | 17 |
| PQ 4 | 353.1 | 214 | - | ADBICA-D9 | 104 | 17 | - |
| PQ 4 | 345.2 | 215 | 300.2 | ADB-PINACA | 94 | 13 | 25 |
| PQ 4 | 354.1 | 224.2 | - | ADB-PINACA | 83 | 25 | - |
| PQ 4 | 360.2 | 155 | 127 | AM2201 | 150 | 25 | 57 |
| PQ 4 | 365.2 | 135.1 | 93.2 | APICA | 138 | 29 | 57 |
| PQ 4 | 366.2 | 135.1 | 93.2 | APINACA (aka AKB-48) | 95 | 25 | 57 |
| PQ 4 | 336.1 | 135.1 | - | APINACA-D9 (aka AKB-48-D9) | 68 | 21 | - |
| PQ 4 | 404.2 | 135.1 | 93.2 | FUB-AKB-48 | 94 | 21 | 61 |
| PQ 4 | 361.2 | 127 | 155 | FUBIMINA | 148 | 33 | 61 |
| PQ 4 | 397.1 | 252 | 109.1 | FUB-PB-22 | 99 | 9 | 41 |
| PQ 4 | 342.2 | 155 | 127 | JWH-018 | 165 | 25 | 57 |
| PQ 4 | 351.2 | 155 | - | JWH-018-D9 | 122 | 25 | - |
| PQ 4 | 371.2 | 354.2 | 241 | MAB-CHMINACA | 110 | 5 | 29 |
| PQ 4 | 375.2 | 245 | - | MAB-CHMINACA-D4 | 83 | 29 | - |
| PQ 4 | 386.2 | 241.1 | 145 | MDMB-CHMINACA | 113 | 25 | 45 |
| PQ 4 | 376.2 | 232 | 144 | NM2201 | 83 | 9 | 45 |
| PQ 4 | 359.2 | 214 | 144 | PB-22 | 95 | 9 | 41 |
| PQ 4 | 343.2 | 215 | 145 | THJ018 | 133 | 13 | 37 |
| PQ 4 | 312.2 | 125 | 55.3 | UR-144 | 150 | 21 | 45 |
| PQ 4 | 317.1 | 125.1 | - | UR-144-D5 | 117 | 25 | - |
| PQ 4 | 312.2 | 214 | 144 | UR-144 Degradant | 123 | 21 | 41 |
| PQ 4 | 330.2 | 125.1 | 55.3 | XLR-11 | 150 | 21 | 49 |
| PQ 4 | 335.1 | 125.1 | - | XLR-11-D5 | 107 | 25 | - |
| PQ 4 | 330.2 | 232.1 | 144 | XLR11 Degradant | 143 | 21 | 45 |
| PQ 4 | 250.1 | 118.1 | 206.1 | 5-Fluoro-PB-22-(3-carboxyindole) | 99 | 9 | 21 |
| PQ 4 | 255.1 | 123.1 | - | 5-Fluoro-PB-22-(3-carboxyindole)-D5 | 87 | 25 | - |
| PQ 4 | 373.2 | 356.2 | 257 | AB-CHMINACA M1A | 100 | 5 | 25 |
| PQ 4 | 374.2 | 257 | 145 | AB-CHMINACA M3A | 110 | 21 | 41 |
| PQ 4 | 263.2 | 245 | - | AB-CHMINACA M4-D4 | 99 | 13 | - |
| PQ 4 | 347.2 | 330.1 | 41.3 | AB-PINACA-(5-hydroxypentyl) | 81 | 5 | 73 |
| PQ 4 | 372.2 | 155 | 127.1 | JWH-018-pentanoic acid | 128 | 25 | 61 |
| PQ 4 | 376.2 | 155 | - | JWH-018-pentanoic acid-D4 | 157 | 25 | - |
| PQ 4 | 387.2 | 342.2 | 257 | MAB-CHMINACA M1 | 114 | 13 | 29 |
| PQ 4 | 388.2 | 257 | 145 | MAB-CHMINACA M3 | 112 | 25 | 49 |
| PQ 4 | 328.2 | 125.1 | 55.3 | UR-144-(5-hydroxypentyl) | 150 | 17 | 45 |
| PQ 4 | 333.3 | 125.1 | - | UR-144-(5-hydroxypentyl)-D5 | 128 | 21 | - |
| PQ 4 | 342.2 | 125.1 | 55.3 | UR-144-pentanoic acid | 165 | 21 | 49 |
| PQ 4 | 347.2 | 125.1 | - | UR-144-pentanoic acid-D5 | 145 | 21 | - |
| PQ 4 | 342.2 | 244 | 55.3 | UR-144-pentanoic acid degradant | 145 | 21 | 61 |
| PQ 4 | 346.2 | 125.1 | 57.3 | XLR11-(4-hydroxypentyl) | 150 | 21 | 49 |
| PQ 4 | 351.2 | 125.1 | - | XLR11-(4-hydroxypentyl)-D5 | 165 | 25 | - |
